# Tubicolous polychaete worms (Annelida) from Bahía de Chamela Islands Sanctuary, Mexico, with the description of a new bamboo worm

**DOI:** 10.3897/BDJ.8.e57572

**Published:** 2020-09-16

**Authors:** Beatriz Yáñez-Rivera, María Ana Tovar-Hernández, Cristian Moisés Galván-Villa, Eduardo Ríos-Jara

**Affiliations:** 1 CONACYT- Centro de Investigación en Alimentación y Desarrollo A.C. (CIAD), Unidad Mazatlán en Acuicultura y Manejo Ambiental, Mazatlán, Mexico CONACYT- Centro de Investigación en Alimentación y Desarrollo A.C. (CIAD), Unidad Mazatlán en Acuicultura y Manejo Ambiental Mazatlán Mexico; 2 Universidad Autónoma de Nuevo León, Facultad de Ciencias Biológicas, Laboratorio de Biosistemática, San Nicolás de los Garza, Nuevo León, Mexico Universidad Autónoma de Nuevo León, Facultad de Ciencias Biológicas, Laboratorio de Biosistemática San Nicolás de los Garza, Nuevo León Mexico; 3 Universidad de Guadalajara, Centro Universitario de Ciencias Biológicas y Agropecuarias, Departamento de Ecología, Zapopan, Mexico Universidad de Guadalajara, Centro Universitario de Ciencias Biológicas y Agropecuarias, Departamento de Ecología Zapopan Mexico

**Keywords:** Polychaeta, Sedentaria, *
Clymenura
*, Western Mexico, Tropical Eastern Pacific.

## Abstract

**Background:**

The islands and islets of Bahía de Chamela, in the Eastern Tropical Pacific, were declared as the first marine sanctuary in Mexico and has been protected since 2002. Their marine biodiversity has been documented in a series of papers in the last decade, but only three species of polychaete worms have been reported.

**New information:**

Sixteen species of sedentary polychaete worms belonging to the families Maldanidae, Oweniidae, Sabellariidae, Sabellidae and Serpulidae are reported to the Bahía de Chamela Islands Sanctuary, 15 of these species constituting the first records in the area. *Isocirrus tropicus* (Monro, 1928) (Monro 1928) and *Notaulax californica* (Treadwell, 1906) (Treadwell 1906) constitute new records to Mexico; *Idanthyrsus mexicanus* Kirtley, 1904 (Kirtley 1994) is first recorded since its description and one species of bamboo worm (Maldanidae) is described as new to science. The new species belongs to the genus *Clymenura* Verril, 1900 (Verrill 1900) and its characterised by the presence of a glandular shield on chaetiger 8; a cephalic plaque oval with smooth margins and a rounded palpode; nuchal organs straight, parallel, almost full length of plaque; manubriavicular uncini present from chaetiger 1 with 3–4 teeth above the main fang without hairs or bristles; two pre-anal achaetous segments with tori; an anal funnel with alternating triangular cirri, being the longest that are located mid-ventrally.

## Introduction

The islands and islets of Bahía de Chamela, in the Eastern Tropical Pacific, were declared as the first marine sanctuary in Mexico and has been protected since 2002 (Miranda et al. 2011). Bahía de Chamela presents a high environmental heterogeneity, in which there is a considerable number of species (López-Uriarte et al. 2009), making it a priority site of extreme importance for the conservation of its marine ecosystems (CONABIO-CONANP-TNC-PRONATURA 2007). In addition, it is an area of fishing importance and tourist potential for the Jalisco coast.

The marine biodiversity of Bahía de Chamela has been documented in a series of papers in the last decade: macroinvertebrates (López-Uriarte et al. 2009), hermit crabs (Bastida-Izaguirre et al. 2013), parasitic copepods (Morales-Serna et al. 2013), mud shrimps (Ayón-Parente et al. 2013), caprellid shrimps (Galván-Villa and Ayón-Parente 2015), caridean shrimps (Ayón-Parente et al. 2016), echinoderms (Ríos-Jara et al. 2013, Galván-Villa et al. 2018, Solís-Marín et al. 2018), lancelets (Galván-Villa et al. 2017) and fishes (Galván-Villa et al. 2016). However, only three species of polychaete worms have been reported in Bahía de Chamela: the acoetid *Polyodontes lupinus* (Treadwell 1941, Pettibone 1989), the serpulid *Spirobranchus* sp. and the sabellid *Bispira rugosa* (López-Uriarte et al. 2009).

Polychaetes are segmented worms belonging to the phylum Annelida. They are predominantly marine with some species in fresh and terrestrial groundwaters (Glasby and Timm 2008, Glasby et al. 2009). As many as 11,456 species, 1417 genera and 85 families were recognised as valid up to 2016 (Pamungkas et al. 2019). In Mexico, 1500 species have been reported along their Pacific and Atlantic littorals (Tovar-Hernández et al. 2014). In the present contribution, 16 sedentary polychaete worms are documented to Bahía de Chamela, one of them constitutes a new record to Mexico and one bamboo worm is established as a new species to science, formally described below.

## Materials and methods

### Fieldwork

**Figure 1.**
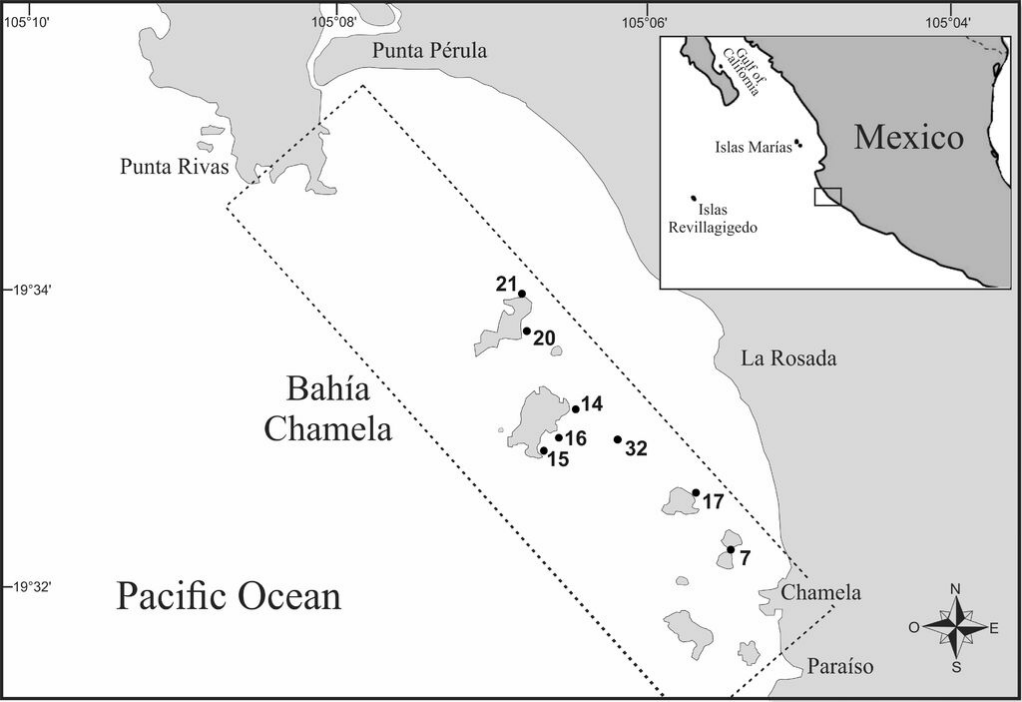
Study area with location of sampling sites and the presence of tubicolous polychaetes.

The material reported in this paper was collected between April 2009 and June 2013 in Bahía de Chamela, Jalisco, Mexico (Fig. 1). The specimens were obtained by hand, snorkelling, scuba diving and using a biological dredge. Worms were fixed in 10% formaldehyde–seawater for 72 h, then excess formalin was removed, specimens were washed with tap water, left in it for 24 h to remove the remaining fixative and sea-water and later transferred to 70% ethanol for long-term preservation.

### Identification

Observations and body measurements were undertaken with a Leica MZ75 stereomicroscope or an Olympus CH30 high power microscope. Photographs were taken with an attached Canon EOS Rebel T7i digital camera. Methyl green and Shirlastain-A were used for improving the contrast of surface features and analysis of the main morphological features.

The new species description is based on the holotype, with variation of paratypes as indicated in parenthesis. Except for the new species here described, descriptions are presented in a broad sense in order that any interested people can follow the identification keys with accuracy. In the nomenclature and taxon discussion sections, systematic contributions are included for those people who require a deeper analysis.

The following taxonomic keys were used: Maldanidae (Salazar-Vallejo and Díaz-Díaz 2009), Oweniidae (Villalobos-Guerrero 2009), Sabellariidae (Bastida-Zavala and Becerrill-Tinoco 2009, Chávez-López 2019), Sabellidae (Tovar-Hernández 2009), Serpulidae (Bastida-Zavala 2009) and specialised literature as indicated in each species sections. Occurrence records are included in Table 1.

Samples were deposited in the following collections: Colección Biológica del Laboratorio de Ecosistemas Marinos y Acuicultura, Universidad de Guadalajara, México (LEMA), Colección Poliquetológica from Universidad Autónoma de Nuevo León, México (UANL) and Colección Regional de Invertebrados Marinos, Instituto de Ciencias del Mar y Limnología, Universidad Nacional Autónoma de México (ICML–EMU).

## Taxon treatments

### Clymenura scutata

Tovar-Hernández and Yáñez-Rivera, 2020 sp. n.

043A950F-89BA-54CB-8CE2-1C179A8A8673

642B9856-331A-46F0-A1BA-59700A4D490A

#### Materials

- **Type status:** Holotype. **Occurrence:** catalogNumber: LEMA-PO153; recordedBy: Beatriz Yáñez-Rivera; lifeStage: Adult; **Taxon:** phylum: Annelida; class: Polychaeta; family: Maldanidae; genus: Clymenura; **Location:** higherGeographyID: Pacific Ocean; higherGeography: Tropical Eastern Pacific; continent: America; islandGroup: Islas de Chamela; island: Isla Cocinas; country: México; countryCode: MX; stateProvince: Jalisco; municipality: La Huerta; maximumDepthInMeters: 7; verbatimLatitude: 19°32’57’’N; verbatimLongitude: 105°06’20’’W; **Identification:** identifiedBy: María Ana Tovar-Hernández; **Event:** samplingProtocol: Scuba dive; eventDate: June 27, 2013; year: 2013; month: 6; day: 27; habitat: On rock; fieldNumber: Site 16; **Record Level:** language: Spanish; institutionID: Universidad de Guadalajara; collectionID: Colección Biológica del Laboratorio de Ecosistemas Marinos y Acuicultura, Universidad de Guadalajara, México; institutionCode: UDG; collectionCode: LEMA
- **Type status:** Paratype. **Occurrence:** catalogNumber: UANL 8144; recordedBy: Beatriz Yáñez-Rivera; individualCount: 3; lifeStage: Adult; **Taxon:** phylum: Annelida; class: Polychaeta; family: Maldanidae; genus: Clymenura; **Location:** higherGeographyID: Pacific Ocean; higherGeography: Tropical Eastern Pacific; continent: America; islandGroup: Islas de Chamela; island: Isla Cocinas; country: México; countryCode: MX; stateProvince: Jalisco; municipality: La Huerta; maximumDepthInMeters: 7; verbatimLatitude: 19°32’57’’N; verbatimLongitude: 105°06’20’’W; **Identification:** identifiedBy: María Ana Tovar-Hernández; **Event:** samplingProtocol: Scuba dive; eventDate: June 27, 2013; year: 2013; month: 6; day: 27; habitat: On rock; fieldNumber: Site 16; **Record Level:** language: Spanish; institutionID: Universidad Autónoma de Nuevo León; collectionID: UANL, NL INV 0002-05-09; institutionCode: UANL; collectionCode: UANL
- **Type status:** Paratype. **Occurrence:** catalogNumber: ICML–EMU–12758; recordedBy: Beatriz Yáñez-Rivera; individualCount: 1; lifeStage: Adult; **Taxon:** phylum: Annelida; class: Polychaeta; family: Maldanidae; genus: Clymenura; **Location:** higherGeographyID: Pacific Ocean; higherGeography: Tropical Eastern Pacific; continent: America; islandGroup: Islas de Chamela; island: Isla Cocinas; country: México; countryCode: MX; stateProvince: Jalisco; municipality: La Huerta; maximumDepthInMeters: 8; verbatimLatitude: 19°33’07’’N; verbatimLongitude: 105°06’31’’W; **Identification:** identifiedBy: María Ana Tovar-Hernández; **Event:** samplingProtocol: Dredge; eventDate: June 27, 2013; year: 2013; month: 6; day: 27; habitat: On rock; fieldNumber: Site 14; **Record Level:** language: Spanish; institutionID: Universidad Nacional Autónoma de México, Instituto de Ciencias del Mar y Limnología; collectionID: Colección Regional de Invertebrados Marinos; institutionCode: UNAM-ICML; collectionCode: ICML-EMU

#### Description

**Figure 2.**
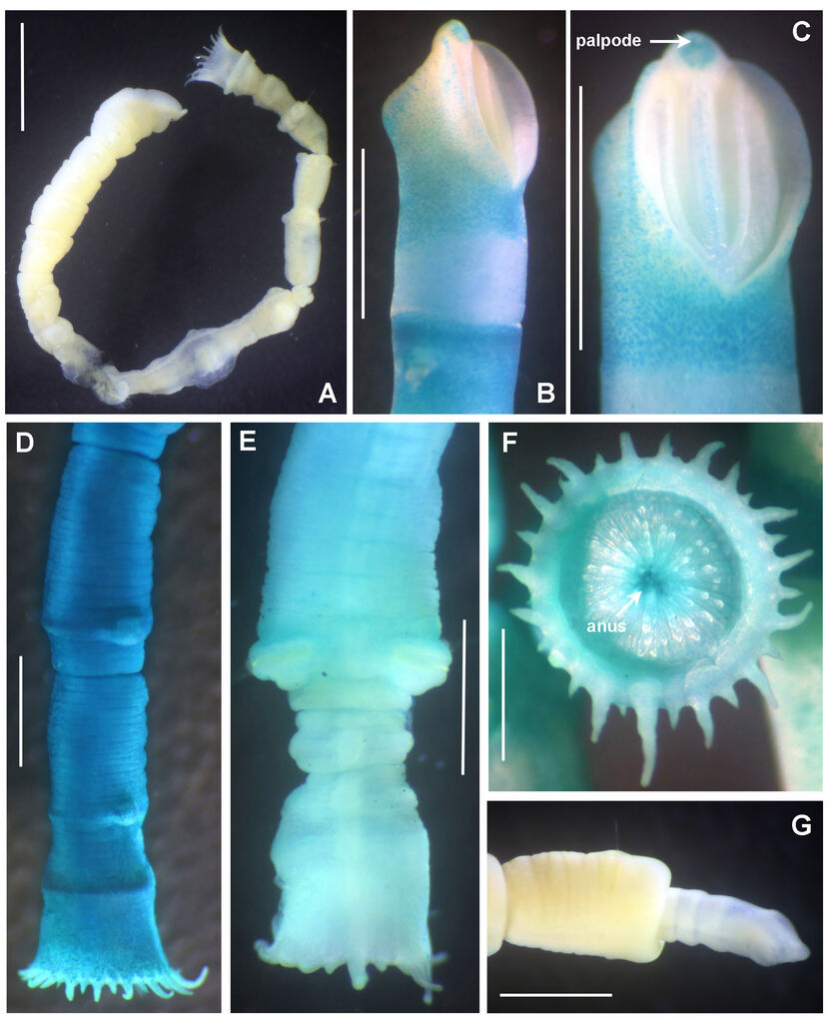
*Clymenura scutata* sp. n. A) Entire worm, lateral view, tube removed, B–C) cephalic plaque, latero-dorsal views, D–E) posterior end and anal plaque, lateral and ventral view, respectively, F) anal plaque, posterior view, G) worm regenerating cephalic plaque and first anterior segments. A–D, F) Holotype LEMA-PO153, E, G) Paratype UANL 8144. Scale bars: A) 2 mm, B–E, G) 1 mm, F) 0.6 mm.

**Figure 3.**
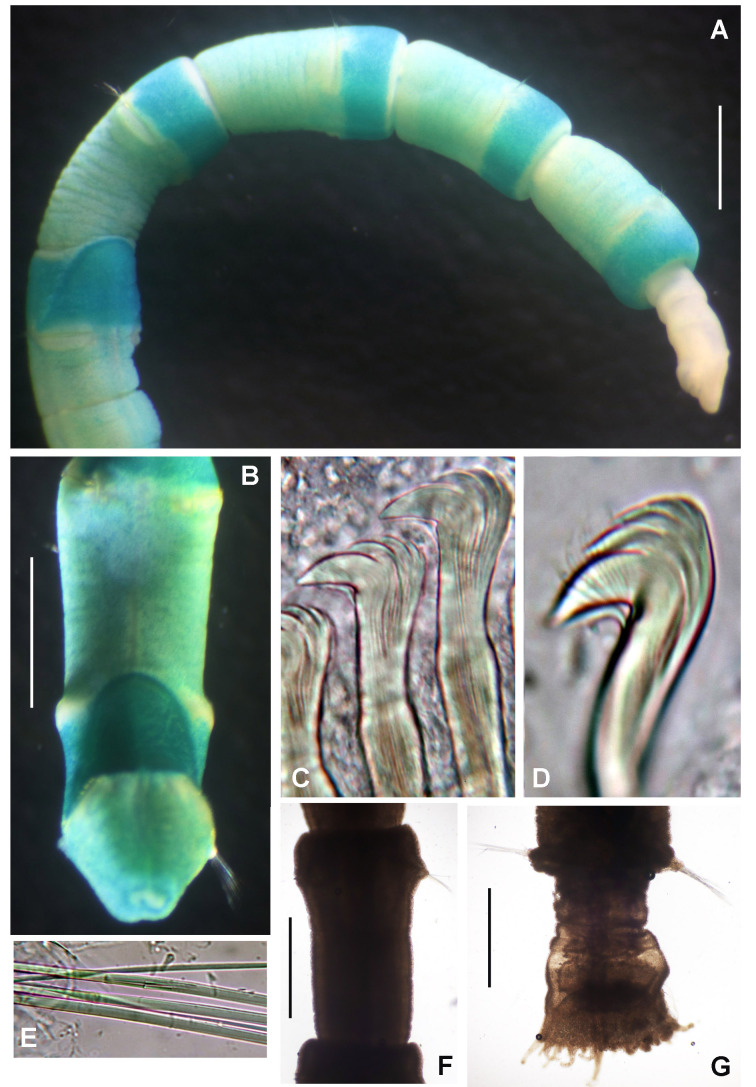
*Clymenura scutata* sp. n. A) Worm with cephalic plaque and first three chaetigers under regeneration, B) glandular shield in chaetiger 8, C) manubriavicular uncini from 1st chaetiger, D) manubriavicular uncinus from 11 chaetiger, E) notochaetae from 11 chaetiger, F) third chaetiger, G) last chaetiger. A, G) Paratype UANL 8144, B–F) Holotype LEMA-PO153. Scale bars: A–B) 1 mm, C–E) 1000X magnification, F-G) 0.5 mm.

Preserved holotype complete, with body pale without any distinctive pigmentation pattern (Fig. 2A). Body 32 mm length (complete paratypes: 28 mm in paratype 2 (UANL-8144), 20 mm in paratype 4 (ICML-EMU-12758); incomplete paratypes: 28 mm in paratype 1 (UANL-8144), 10 mm in paratype 3 (UANL-8144); 1 mm width (1 mm in paratypes 2 (UANL-8144) and 4 (ICML-EMU-12758); 0.5 mm in paratype 2 (UANL-8144), 0.8 mm in paratype 3 (UANL-8144). Body with 14 chaetigers (complete paratypes: 20 chaetigers in paratype 2 (UANL-8144); 15 chaetigers in paratype 4 (ICML-EMU-12758), two preanal achaetigerous segments with tori (two preanal achaetigers segments in all paratypes, except in paratype 1 (UANL-8144) whose posterior end is missing). Well-developed cephalic plaque, oval, with lateral margins smooth, entire (no incisions or notches) (Fig. 2B–C) and posterior margin V-shaped (Fig. 2C). Nuchal organs straight, parallel, almost full length of plaque (Fig. 2C). Palpode well developed with rounded tip (Fig. 2C). No ocelli. No segmental collars (Fig. 2A, Fig. 3A). Ventral shield of chaetiger 8 half-oval shaped, revealed with methyl green (Fig. 3A–B). Chaetigers 11 and 12 are the longest (paratype 1, UANL-8144: chaetigers 10 and 11; paratype 2, UANL-8144: chaetigers 13–15). Notochaetae capillaries of one type, smooth by only slightly winged (Fig. 3E); those from anterior segments short (Fig. 3F), whereas those from posterior chaetigers as long was two times the length of capillaries from anterior segments (Fig. 3G). Single row of manubriavicular uncini present, starting in chaetiger 1 with 3–4 small teeth above the main fang, subrostral bristles or hairs or barbules not seen (Fig. 3C) and long and nearly straight handles. Manubriavicular uncini from median and posterior chaetigers similar shape to those present in chaetiger 1, but with developed subrostral bristles or hairs or barbules (Fig. 3D) and handles curved. First and second preanal segments about 1/2 and 2/3 length of last chaetigerous segment, respectively, with achaetous reduced torus (Fig. 2A–B). Pygidial funnel well developed (Fig. 2D–F), ending with 28 distinct triangular cirri (26–29 in paratypes), well separated and alternated short with long (twice longer than short ones) and with the ventral cirrus long (about twice the length of the long alternates) (Fig. 2A–B). Pygidial funnel with a distinctive callus ring (Fig. 2A). Anus terminal, located just in the centre of the funnel (Fig. 2F). Glandular pattern revealed with methyl green shows blue bands in anterior segments (Fig. 3A) and glandular shield on chaetiger 8 stains deep blue (Fig. 3B).

#### Diagnosis

A worm of 32 mm length, 1 mm width, with 14 chaetigers, two pre-anal achaetous segments with tori, manubriavicular uncini present from chaetiger 1 with 3–4 teeth above the main fang without barbules; manubriavicular uncini similar to that of chaetiger 1, but the number of barbules increases towards the most posterior segments. Cephalic plaque oval with entire, smooth margins; rounded palpode; nuchal organs straight, parallel, almost full length of plaque. No ocelli, no segmental collars. Anal funnel present with 28 distinct alternating triangular cirri: short and long (twice longer than short ones) and with the mid-ventral cirrus longest (about twice the length of the adjacent long). Chaetiger 8 glandular shield extending anteriorly and ventrally from notochaetal fascicle forming a half-oval.

#### Etymology

From the Latin *scutata*, meaning armed with a shield and referring to the glandular shield on chaetiger 8.

#### Taxon discussion

The specimens, here reviewed, match with the emendation of *Clymenura* provided by Read (2011). The genus *Clymenura* has never been reported in Mexico. The closest record to the Mexican Pacific of a *Clymenura* is *C. gracilis* Hartman, 1969 (Hartman 1969), a species originally described from Santa Monica, California. However, Read (2011) considers *C. gracilis* as *incertae sedis* because the holotype lacks of ventral shield on chaetiger 8; consequently, it cannot be a member of *Clymenura*.

Amongst the nine valid taxa of *Clymenura* worldwide, two species have been described from America: *C. cirrata* (Ehlers, 1887 as *Clymene*) (Ehlers 1887) from Florida and *C. columbiana* (Berkeley, 1929 as *Leichone*) (Berkeley 1929) to western Canada (Table 2). *Clymenura scutata* sp. n., and *C. columbiana* have an anal plaque with a long mid-ventral cirrus; however, *Clymenura scutata* sp. n. differs from *C. columbiana* by:

1. lateral margins of the cephalic plaque are entire,
2. does not present prostomial ocelli,
3. it has a marked dentition of uncini in first thoracic chaetiger and
4. it has two preanal achaetous segments (cephalic plaque notched laterally, prostomial eyes present, reduced or reminiscent dentition of uncini in first thoracic chaetiger and three pre-anal achaetous segments in *C. columbiana*).

*Clymenura cirrata* is characterised by the presence of four long anal cirri (only the mid-ventral is long in *Clymenura scutata* sp. n.); the lateral margins or the cephalic plaque are notched (entire in *Clymenura scutata* sp. n.); and collars are present in chaetigers 2–4 (absent in *Clymenura scutata* sp. n.).

The rest of the species in the genus were described from high north European latitudes and the Northern Pacific region: Novaya Zemlya (one species), Japan (three species), Norway (one species), the Laptev Sea (one species), except for *Clymenura snaiko* Read, 2011 (Read 2011), that was described from New Zealand (see Read 2011 to further details). *Clymenura snaiko* and *Clymenura scutata* sp. n., have the mid-ventral anal cirrus long, but the Mexican species have alternated cirri, whereas all other cirri are similar in length in *C. snaiko*. In addition, the lateral margins of the cephalic plaque are notched in *C. snaiko* versus entire in *C. scutata* sp. n.

Referring to intraspecific variation, a paratype was found under regeneration of the anterior end of the body (Fig. 2G). The cephalic plaque and the first three segments are a half narrower than segment 4 and those subsequent. However, the glandular shield on chaetiger 8 is present, it maintain the half-oval shape. As the number of chaetigers (and size) varies in all paratypes, their use, therefore, cannot be considered diagnostic.

### Owenia collaris

Hartman, 1955

8E4CF95B-2657-55A2-B348-2F9DDFA22FDD

Owenia fusiformis collaris in *Hartman 1955*: 46, pl. 2, figs. 6–7.— Hartman 1963: 70.
Owenia collaris Hartman 1969: 493–494, figs 1-4.— Kudenov 1975b: 225.-- Blake 2000: 120–121, fig. 5.9.

#### Materials

- **Type status:** Other material. **Occurrence:** catalogNumber: LEMA-PO155; recordedBy: Beatriz Yáñez-Rivera; individualCount: 2; **Taxon:** phylum: Annelida; class: Polychaeta; order: Sabellida; family: Oweniidae; genus: Owenia; **Location:** higherGeographyID: Pacific Ocean; higherGeography: Tropical Eastern Pacific; continent: America; islandGroup: Islas de Chamela; island: Isla La Colorada; country: México; countryCode: MX; stateProvince: Jalisco; municipality: La Huerta; maximumDepthInMeters: 6; verbatimLatitude: 19°32’23’’N; verbatimLongitude: 105°05’31’’W; **Identification:** identifiedBy: María Ana Tovar-Hernández; **Event:** samplingProtocol: Dredge; eventDate: June 26, 2013; year: 2013; month: 6; day: 26; habitat: Sand; fieldNumber: Site 17; **Record Level:** language: Spanish; institutionID: Universidad de Guadalajara; collectionID: Colección Biológica del Laboratorio de Ecosistemas Marinos y Acuicultura, Universidad de Guadalajara, México; institutionCode: UDG; collectionCode: LEMA

#### Description

Entire worms with 14–18 segments, 12–14 mm length, 0.4–0.7 mm width, tentacular crown 0.4–0.7 mm length. Prostomium with a short tentacular crown consisting of 8–12 basal branched trunks (Fig. 5A–E). Anterior border of peristomium with brownish spots (Fig. 5D–E). Eyes absent. First three chaetigers of thorax with notochaetae only, chaetiger 3 reduced, with notochaetae shifted dorsally. Segment 5 longest (Fig. 5G). Dorsal glandular ridges absent. Dorsal abdominal groove present. Abdominal segments birrameous with capillary notochaetae and neuropodial minute uncini that form into tight bands that nearly encircle the body. These uncini have long shafts with two long, curved teeth situated side by side (Fig. 5F). Pygidium with anal opening positioned dorsally. The largest specimens have oocytes in chaetiger 4.

#### Distribution

Puerto Peñasco, Sonora (Kudenov 1975b) and Chamela Bay (present study).

#### Taxon discussion

Described with ocular spots (Hartman 1955), but considered as absent in the re-description provided by Blake (2000). These spots are present in specimens from Bahía de Chamela (Figs. 5D–E).

### Isocirrus tropicus

(Monro, 1906)

95D09E3A-1483-54BA-A663-4F1697ADACB3

Clymene tropica Monro, 1906 in Monro 1928: 97–98, figs. 17–18.
Isocirrus tropicus .— Salazar-Vallejo 1991: 275 (syntype re-description and relocation).
Euclymene tropica .— Salazar-Vallejo and Díaz-Díaz 2009: 305 (Key).

#### Materials

- **Type status:** Other material. **Occurrence:** catalogNumber: LEMA-PO154; recordedBy: Beatriz Yáñez-Rivera; individualCount: 4; **Taxon:** phylum: Annelida; class: Polychaeta; family: Maldanidae; genus: Isocirrus; **Location:** higherGeographyID: Pacific Ocean; higherGeography: Tropical Eastern Pacific; continent: America; islandGroup: Islas de Chamela; island: Isla Pajarera; country: México; countryCode: MX; stateProvince: Jalisco; municipality: La Huerta; maximumDepthInMeters: 4; verbatimLatitude: 19°33’22’’N; verbatimLongitude: 105°06’50’’W; **Identification:** identifiedBy: María Ana Tovar-Hernández; **Event:** samplingProtocol: Scuba dive; eventDate: June 25, 2013; year: 2013; month: 6; day: 25; habitat: On coral; fieldNumber: Site 21; **Record Level:** language: Spanish; institutionID: Universidad de Guadalajara; collectionID: Colección Biológica del Laboratorio de Ecosistemas Marinos y Acuicultura, Universidad de Guadalajara, México; institutionCode: UDG; collectionCode: LEMA

#### Description

**Figure 4.**
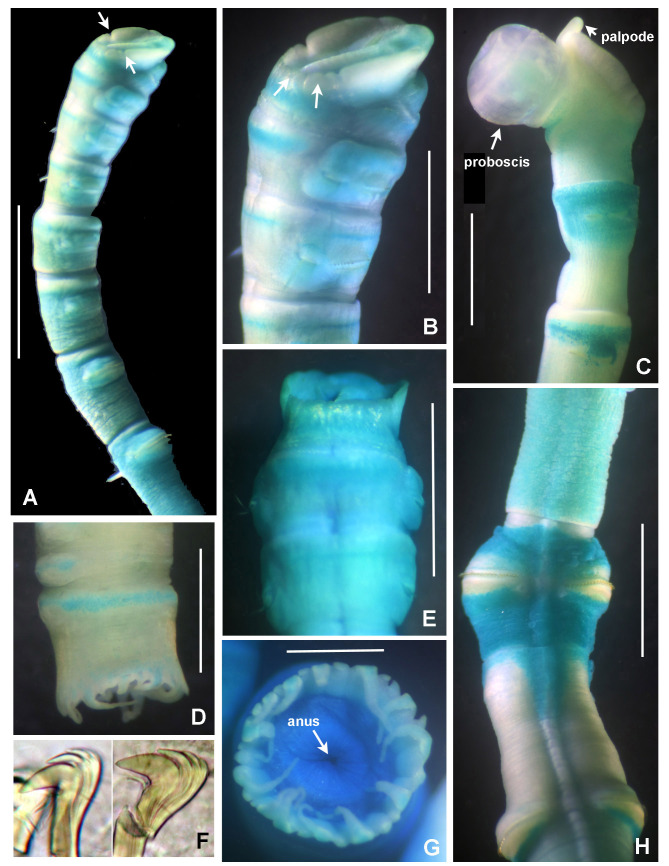
*Isocirrus tropicus* (Monro, 1928). A) Body, anterior part, lateral view, B) cephalic plaque and first two chaetigers, lateral view, C) everted proboscis, D) anal funnel, lateral view, E) dorsal margin of cephalic plaque, F) manubriavicular uncini from 5th chaetiger, G) anal plaque, posterior view, H) mid-ventral whitish ridge starting in chaetiger 7. Scale bars: A) 3 mm, B) 2 mm, C) 1 mm, D) 1.3 mm, E) 1.8 mm, F) 400x magnification, G) 0.5 mm, H) 1.5 mm. Arrows in A pointed to lateral notches of cephalic plaque. In B, arrows pointed to crenulated margin of cephalic plaque.

Incomplete worms 6–36 mm long, 0.5–1.4 mm wide and +12 segments. Cephalic plaque oval with lateral margins notched (Fig. 4A), basal margin with eight shallow crenulations (Fig. 4B, E); digitiform palpod in lateral view (Fig. 4C); nuchal organs straight, parallel, almost full length of plaque. No ocelli, no segmental collars. First segments with acicular spines. Following chaetigers with manubriavicular uncini with three teeth above the main fang with subrostral bristles (hairs or barbules) (Fig. 4F). Ventral mid-line ridge prominent, from segment 7 to end of body (Fig. 4H). One pre-anal achaetous segment with tori. Anal funnel present (Fig. 4D) with 26 distinct alternating triangular cirri: 3–4 long alternated with one short, the longest are 2–3 times the length of short ones (Fig. 4G). Anus terminal (Fig. 4G).

#### Taxon discussion

Originally described from Taboga (Pacific coast of Panama) to 7–9 m depth in sand at low tide (Monro 1928). It was re-described and transferred to *Isocirrus* by Salazar-Vallejo (1991), based on the presence of acicular spines on chaetigers 1–3 and the presence of anal cirri of similar length. According to Salazar-Vallejo (pers. comm. 2020), the inclusion of *Euclymene tropica* in their key was a mistake (Salazar-Vallejo and Díaz-Díaz 2009); it must be included within *Isocirrus*, but having subequal anal cirri.

### Idanthyrsus cretus

Chamberlin, 1919

AA731F21-8B42-58D4-A7BA-D733378F1EBB

Idanthyrsus cretus Chamberlin, 1919 Chamberlin 1919: 485–487, pt. 76, figs. 8–15.
Idanthyrsus pennatus (not Peters 1854).— Monro 1933: 1063, fig. 13.— Rioja 1942: 155–157, figs. 1–14.— Hartman 1944a: 336, pt. 31, fig. 35.— Berkeley and Berkeley 1958: 405.— Rioja 1959: 255.— Rioja 1962: 199.— Fauchald 1977: 54.— Salazar-Vallejo et al. 1990: 213, fig. 6.— Bastida-Zavala 1993: 12, 14, 32.— Bastida-Zavala 1995: 22.
Idanthyrsus cretus .— Kirtley 1994: 95–96, fig. 6.6.— Gómez et al. 1997: 1070.— Hernández-Alcántara et al. 2003: 9.— Chávez-López and Cruz-Gómez 2019: 5, 8, fig. 2E.— Chávez-López 2019: 21–28, figs. 2H–I, K, 5–6.

#### Materials

- **Type status:** Other material. **Occurrence:** catalogNumber: LEMA-PO156; recordedBy: Beatriz Yáñez-Rivera; individualCount: 1; **Taxon:** phylum: Annelida; class: Polychaeta; order: Sabellida; family: Sabellariidae; genus: Idanthyrsus; **Location:** higherGeographyID: Pacific Ocean; higherGeography: Tropical Eastern Pacific; continent: America; islandGroup: Islas de Chamela; island: Isla Pajarera; country: México; countryCode: MX; stateProvince: Jalisco; municipality: La Huerta; maximumDepthInMeters: 4; verbatimLatitude: 19°33’22’’N; verbatimLongitude: 105°06’50’’W; **Identification:** identifiedBy: María Ana Tovar-Hernández; **Event:** samplingProtocol: Scuba dive; eventDate: June 25, 2013; year: 2013; month: 6; day: 25; habitat: On rock; fieldNumber: Site 21; **Record Level:** language: Spanish; institutionID: Universidad de Guadalajara; collectionID: Colección Biológica del Laboratorio de Ecosistemas Marinos y Acuicultura; institutionCode: UDG; collectionCode: LEMA
- **Type status:** Other material. **Occurrence:** catalogNumber: LEMA-PO157; recordedBy: Beatriz Yáñez-Rivera; individualCount: 4; **Taxon:** phylum: Annelida; class: Polychaeta; order: Sabellida; family: Sabellariidae; genus: Idanthyrsus; **Location:** higherGeographyID: Pacific Ocean; higherGeography: Tropical Eastern Pacific; continent: America; islandGroup: Islas de Chamela; island: Isla Pajarera; country: México; countryCode: MX; stateProvince: Jalisco; municipality: La Huerta; maximumDepthInMeters: 4; verbatimLatitude: 19°33’29’’N; verbatimLongitude: 105°06’40’’W; **Identification:** identifiedBy: María Ana Tovar-Hernández; **Event:** samplingProtocol: Snorkel; eventDate: June 27, 2013; year: 2013; month: 6; day: 27; habitat: On coral; fieldNumber: Site 2; **Record Level:** language: Spanish; institutionID: Universidad de Guadalajara; collectionID: Colección Biológica del Laboratorio de Ecosistemas Marinos y Acuicultura; institutionCode: UDG; collectionCode: LEMA

#### Description

Gregarious worms commonly known as “honey comb worms”. Tubes constructed with sea shells fragments, echinoderm spines debris, sand and small gravel. Complete specimens 13–29 mm long, 2–4 mm wide, with 22–31 abdominal chaetigers and a caudal peduncle 3–5 mm long. Body divided into four specialised regions: operculum, parathorax (with three segments), abdomen and caudal region (Fig. 6A–B). Operculum composed of a crown and a peduncle, forming two not fused lobes (Fig. 6E). Outer paleae with blades curved distally and lateral denticles curved (Fig. 6G). Inner paleae with straight blades, smooth and blunt tips (Fig. 6H). A pair of nuchal hooks with limbation or hood below the concave area (Fig. 6F). Uncini with six pairs of teeth (Fig. 6C–D).

#### Distribution

*Idanthyrsus cretus* is a widely-reported species from Isla Cedros (Baja California, Mexico) to Ecuador, including the Galapagos Islands (Bastida-Zavala and Becerrill-Tinoco 2009).

#### Taxon discussion

Detailed description and illustrations are available in Kirtley (1994).

### Idanthyrsus mexicanus

Kirtley, 1994

2563BE61-B619-5483-AFF6-293D6480FF22

Idanthyrsus mexicanus in *Kirtley 1994*: 105–106, fig. 6.12.

#### Materials

- **Type status:** Other material. **Occurrence:** catalogNumber: LEMA-PO158; recordedBy: Beatriz Yáñez-Rivera; individualCount: 6; **Taxon:** phylum: Annelida; class: Polychaeta; order: Sabellida; family: Sabellariidae; genus: Idanthyrsus; **Location:** higherGeographyID: Pacific Ocean; higherGeography: Tropical Eastern Pacific; continent: America; islandGroup: Islas de Chamela; island: Isla Pajarera; country: México; countryCode: MX; stateProvince: Jalisco; municipality: La Huerta; maximumDepthInMeters: 7; verbatimLatitude: 19°33’22’’N; verbatimLongitude: 105°06’50’’W; **Identification:** identifiedBy: María Ana Tovar-Hernández; **Event:** samplingProtocol: Scuba dive; eventDate: June 26, 2013; year: 2013; month: 6; day: 26; habitat: On rock-coral; fieldNumber: Site 21; **Record Level:** language: Spanish; institutionID: Universidad de Guadalajara; collectionID: Colección Biológica del Laboratorio de Ecosistemas Marinos y Acuicultura; institutionCode: UDG; collectionCode: LEMA

#### Description

Gregarious worms commonly known as “honey comb worms”. Tubes constructed with sea shells fragments, echinoderm spines debris, sand and small gravel (as well as *I. cretus*). Complete specimens 8–11.1 mm long, 1–2 mm wide, with 27–30 abdominal chaetigers and a caudal peduncle 2–3 mm long. Body divided into four specialised regions: operculum, parathorax (with three segments), abdomen and caudal region (Fig. 7A). Operculum composed by a crown and a peduncle, forming two not fused lobes. Median organ short, not longer than opercular lobes. Outer palea with straight blades; base of blades with transversal thecal fringes (Fig. 7E), lateral denticles straight (Fig. 7F), longer and narrower towards tips (Fig. 7G). Inner paleae straight with markedly, transversal thecae and pointed tips (Fig. 7H–J). A pair of nuchal hooks curved (Fig. 7B) without hoods or limbations on concave sides of hooks (Fig. 7C). Abdominal uncini with six pairs of teeth in side view (Fig. 7D).

#### Distribution

This constitutes the fist record since its establishment by Kirtley (1994).

#### Taxon discussion

This species was described from Bahía Tenacatita, west of the islets off Barra de Navidad, between 45.7 and 64 m depth. A second species from Western Mexico is *Idanthyrsus armatopsis* Fauchald, 1972 Fauchald (1972) described from bathyal depths (1200 -1400 m) in the Gulf of California. *Idanthyrsus mexicanus* and *I. armatopsis* shares the presence of nuchal hooks without hoods, but the inner paleae in *I. mexicanus* have pointed tips versus blunt tips in *I. armatopsis.* Number of teeth of abdominal uncini was not described by Kirtley (1994) to *I. mexicanus*, but specimens here reported from Chamela Bay have six teeth in side view versus 7-8 teeth in *I. armatopsis*. The median organ in *I. mexicanus* (not described in original description by Kirtley 1994) is short, extending only 1/4 above lateral opercular lobes, whereas in *I. armatopsis*, this organ extends 1/2 above lateral opercular lobes.

### Idanthyrsus

sp.

188ABD5B-417D-5567-A794-B743317C4EAF

Idanthyrsus sp. considered as new to science by Chávez-López 2019: 37–45, figs 9–10.

#### Materials

- **Type status:** Other material. **Occurrence:** catalogNumber: LEMA-PO159; recordedBy: Irving Ramírez-Santana; individualCount: 6; **Location:** higherGeographyID: Pacific Ocean; higherGeography: Tropical Eastern Pacific; continent: America; islandGroup: Islas de Chamela; island: Isla Cocinas; country: México; countryCode: MX; stateProvince: Jalisco; municipality: La Huerta; minimumDepthInMeters: 3; maximumDepthInMeters: 4; verbatimLatitude: 19°32’57’’N; verbatimLongitude: 105°06’20’’W; **Identification:** identifiedBy: María Ana Tovar-Hernández; **Event:** samplingProtocol: Snorkel; eventDate: April 23, 2009; year: 2009; month: 4; day: 23; habitat: Rock; fieldNumber: Site 1; **Record Level:** language: Spanish; institutionID: Universidad de Guadalajara; collectionID: Colección Biológica del Laboratorio de Ecosistemas Marinos y Acuicultura; institutionCode: UDG; collectionCode: LEMA

#### Description

Gregarious worms commonly known as “honey comb worms” (as well as *I. cretus and I. mexicanus*). Tubes constructed with sea shells fragments (Fig. 8A). Complete specimens 14.1–14.5 mm long, 1.3–1.5 mm wide, with 26–32 abdominal chaetigers and a caudal peduncle 4–6 mm long. Body divided into four specialised regions: operculum, parathorax (with three segments), abdomen and caudal region where there is a long peduncle (Fig. 8B). Operculum composed by a crown and a peduncle, forming two not fused lobes (Fig. 8C). Outer paleae with straight blades basally, then slightly curved towards tips (Fig. 8F); lateral denticles long, narrow and straight, those from the distal area longer than basal half (Fig. 8F–G). Inner paleae with straight blades, transversal thecae not easily seen and pointed tips (Fig. 8H). Two nuchal hooks with limbate area in concave sides (Fig. 8E). Abdominal uncini with seven teeth in side view (Fig. 8D).

#### Ecology

Found in colony mixed with *Idanthyrsus cretus*.

#### Taxon discussion

Chávez-López (2019) recorded this species to Guerrero and Oaxaca and the formal description of the species is under construction (Chávez-López pers. com. 2020). Specimen from Bahía de Chamela fits the description provided by Chávez-López (2019), except for the presence of inner paleae with pointed tips versus blunt by Chávez-López (2019). Judging from her Figure 9D, we considered that the tip is pointed instead of blunt. In addition, in her Figure 10G, an inner palea from a juvenile has a blunt tip, but it seems broken.

### Acromegalomma circumspectum

(Moore, 1923)

94724949-B94A-574A-A5A6-7863088715EC

Branchiomma circumspectum Moore, 1923 in *Moore 1923*: 239–241, pl. 18, figs. 41–42.
Megalomma circumspectum .— Hartman 1959: 550.— Hartman 1961: 43.— Hartman 1969: 707, figs 1–6.— Rioja 1962: 213–216, figs 145–148.— Fauchald 1972: 33.— Perkins 1984: 363.— Knight-Jones 1997: 314.— Hernández-Alcántara and Solís-Weiss 1999: 29.— Tovar-Hernández and Carrera-Parra 2011: 19–24, figs 4A–J, 6A–V, 28A, G, 29A.
Acromegalomma circumspectum .— Tovar-Hernández et al. 2019: 5, fig. 3A–B.

#### Materials

- **Type status:** Other material. **Occurrence:** catalogNumber: LEMA-PO160; recordedBy: Beatriz Yáñez-Rivera; individualCount: 1; lifeStage: Adult; reproductiveCondition: Ripe; **Taxon:** phylum: Annelida; class: Polychaeta; order: Sabellida; family: Sabellidae; genus: Acromegalomma; **Location:** higherGeographyID: Pacific Ocean; higherGeography: Tropical Eastern Pacific; continent: America; islandGroup: Islas de Chamela; country: México; countryCode: MX; stateProvince: Jalisco; municipality: La Huerta; locality: Canal San Pedro; maximumDepthInMeters: 5; verbatimLatitude: 19°32’01’’N; verbatimLongitude: 105°05’17’’W; **Identification:** identifiedBy: María Ana Tovar-Hernández; **Event:** samplingProtocol: Scuba dive; eventDate: June 26, 2013; year: 2013; month: 6; day: 26; habitat: On rock; fieldNumber: Site 7; **Record Level:** institutionID: Universidad de Guadalajara; collectionID: Colección Biológica del Laboratorio de Ecosistemas Marinos y Acuicultura; institutionCode: UDG; collectionCode: LEMA
- **Type status:** Other material. **Occurrence:** catalogNumber: LEMA-PO161; recordedBy: Beatriz Yáñez-Rivera; individualCount: 3; lifeStage: Adult; reproductiveCondition: Ripe; **Taxon:** phylum: Annelida; class: Polychaeta; order: Sabellida; family: Sabellidae; genus: Acromegalomma; **Location:** higherGeographyID: Pacific Ocean; higherGeography: Tropical Eastern Pacific; continent: America; islandGroup: Islas de Chamela; island: Isla Pajarera; country: México; countryCode: MX; stateProvince: Jalisco; municipality: La Huerta; maximumDepthInMeters: 7; verbatimLatitude: 19°33’22’’N; verbatimLongitude: 105°06’50’’W; **Identification:** identifiedBy: María Ana Tovar-Hernández; **Event:** samplingProtocol: Scuba dive; eventDate: June 26, 2013; year: 2013; month: 6; day: 26; habitat: On rock and coral; fieldNumber: Site 21; **Record Level:** language: Spanish; institutionID: Universidad de Guadalajara; collectionID: Colección Biológica del Laboratorio de Ecosistemas Marinos y Acuicultura; institutionCode: UDG; collectionCode: LEMA

#### Description

Complete specimens 10–19 mm long, 0.8–1.3 mm wide with 8 thoracic chaetigers and 42–61 abdominal chaetigers. Radiolar crown with 13–16 pairs of radioles. Radioles with subdistal eyes in most radioles, spherical. Those from dorsal-most radioles are the largest (Fig. 9A–C_1_), then gradually decreasing their size towards lateral and some ventral radioles (Fig. 9A–B). Ventral-most radioles with ocular spots (lacking ommatidia) (Fig. 9C_2_). Dorsal margins of collar fused to faecal groove. A triangular keel present, projecting ventrally between dorsal lips (Fig. 9E). Dorsal pockets present (Fig. 9E). Length of thoracic tori is the same in all segments and not contacting the lateral margins of ventral shields (Fig. 9D).

#### Taxon discussion

Widely reported in the Gulf of California and Nayarit (Tovar-Hernández and Carrera-Parra 2011), this worm has been found being parasitised by *Gastrodelphys dalesi*, a cyclopoid copepod attached to the radioles, the inner base of the radiolar crown, dorsal lips and attached to the dorsal pockets of collar (Gómez and Tovar-Hernández 2008). A full description and illustrations are available in the revision by Tovar-Hernández and Carrera-Parra (2011).

### Bispira monroi

(Hartman, 1961)

DFAE5AB6-567D-57EF-8FAF-EBBB66E76085

Distylidia monroi Hartman, 1961 in Hartman 1961: 129 (new name for Bispira rugosa var. monterea Monro).
Bispira rugosa monterea .— Kudenov 1975a: 226.
Bispira monroi .— Fauchald 1977: 60–61, figs. 13a–b.— Knight-Jones and Perkins 1998: 446–448, fig. 27.
Bispira rugosa (no Moore).— Kerstitch and Bertsch 2007: 38, fig. 61.— López-Uriarte et al. 2009: 62.

#### Materials

- **Type status:** Other material. **Occurrence:** catalogNumber: LEMA-PO162; recordedBy: Beatriz Yáñez-Rivera; individualCount: 3; lifeStage: Adult; **Taxon:** phylum: Annelida; class: Polychaeta; order: Sabellida; family: Sabellidae; genus: Bispira; **Location:** higherGeographyID: Pacific Ocean; higherGeography: Tropical Eastern Pacific; continent: America; islandGroup: Islas de Chamela; island: Isla Pajarera; country: México; countryCode: MX; stateProvince: Jalisco; municipality: La Huerta; maximumDepthInMeters: 9; verbatimLatitude: 19°33’22’’N; verbatimLongitude: 105°06’50’’W; **Identification:** identifiedBy: María Ana Tovar-Hernández; **Event:** samplingProtocol: Scuba dive; eventDate: June 27, 2013; year: 2013; month: 6; day: 27; habitat: On rock; fieldNumber: Site 21; **Record Level:** language: Spanish; institutionID: Universidad de Guadalajara; collectionID: Colección Biológica del Laboratorio de Ecosistemas Marinos y Acuicultura; institutionCode: UDG; collectionCode: LEMA

#### Description

Body 13–34 mm long, 2–4 mm wide, radiolar crown 6–17 mm long with 13–29 pairs of radioles, thorax with 13 chaetigers and abdomen with 46–82 chaetigers. Gregarious species (Fig. 10A). Soft tubes constructed by fine sand. Each radiole with three purple bands without a uniform distribution pattern (Fig. 10B). Within each purple radiolar band, there is a pair of compound eyes (three pairs of eyes per radiole). Ventral margins of thick base spiralling inwards to form three whorls (Fig. 10C). Collar with purple spots dorsally and ventrally. Thorax with purple spots anterior to each thoracic notochaetae (Fig. 10D). Purple maculae between ventral shields and tori (Fig. 10E). In lateral view, dorsal and ventral spots are seen in each segment, located at the ends of notochaetae and neurochaetae (Fig. 10F).

#### Taxon discussion

This is the only fan worm that has been included in the invertebrate guides from the Gulf of California (Kerstitch and Bertsch 2007). It has been reported in the Gulf of California (Kudenov 1975a) and Pacific Panama and Costa Rica (Knight-Jones and Perkins 1998).

### Chone mollis

(Bush, 1904)

417A3B7E-3302-5B81-9B69-6B49F84FBB8C

Metachone mollis in Bush 1904: 216, pt. 35, figs. 19–20, 28.
Chone mollis .— Hartman 1942: 87, figs. 141–143; Hartman 1944b: 279; Hartman 1969: 673; Banse 1972: 469, fig. 3.— Tovar‐Hernández 2007: 539–543, figs. 10, 19B.

#### Materials

- **Type status:** Other material. **Occurrence:** catalogNumber: LEMA-PO163; recordedBy: Beatriz Yáñez-Rivera; individualCount: 2; **Taxon:** phylum: Annelida; class: Polychaeta; order: Sabellida; family: Sabellidae; genus: Chone; **Location:** higherGeographyID: Pacific Ocean; higherGeography: Tropical Eastern Pacific; continent: America; islandGroup: Islas de Chamela; island: Isla Cocinas; country: México; countryCode: MX; stateProvince: Jalisco; municipality: La Huerta; maximumDepthInMeters: 8; verbatimLatitude: 19°33’06’’N; verbatimLongitude: 105°06’43’’W; **Identification:** identifiedBy: María Ana Tovar-Hernández; **Event:** samplingProtocol: Dredge; eventDate: June 27, 2013; year: 2013; month: 6; day: 27; habitat: Sand-rubble; fieldNumber: Site 3; **Record Level:** language: Spanish; institutionID: Universidad de Guadalajara; collectionID: Colección Biológica del Laboratorio de Ecosistemas Marinos y Acuicultura; institutionCode: UDG; collectionCode: LEMA
- **Type status:** Other material. **Occurrence:** catalogNumber: LEMA-PO164; recordedBy: Beatriz Yáñez-Rivera; individualCount: 1; **Taxon:** phylum: Annelida; class: Polychaeta; order: Sabellida; family: Sabellidae; genus: Chone; **Location:** higherGeographyID: Pacific Ocean; higherGeography: Tropical Eastern Pacific; continent: America; islandGroup: Islas de Chamela; island: Isla La Colorada; country: México; countryCode: MX; stateProvince: Jalisco; municipality: La Huerta; maximumDepthInMeters: 6; verbatimLatitude: 19°32’23’’N; verbatimLongitude: 105°05’31’’W; **Identification:** identifiedBy: María Ana Tovar-Hernández; **Event:** samplingProtocol: Dredge; eventDate: June 26, 2013; year: 2013; month: 6; day: 26; habitat: Sand-rubble; fieldNumber: Site 17; **Record Level:** language: Spanish; institutionID: Universidad de Guadalajara; collectionID: Colección Biológica del Laboratorio de Ecosistemas Marinos y Acuicultura; institutionCode: UDG; collectionCode: LEMA

#### Description

Body 15 mm long, 1.4 mm wide, radiolar crown 3.2 mm long with 12 pairs of radioles, thorax with eight chaetigers and abdomen with 30 chaetigers. Solitary fan worm from soft bottoms. Branchial crown cone-shaped when it is open. Radioles united by a long palmate membrane that occupies 3/4 of their length (Fig. 11A, C). Radioles with broad flanges (Fig. 11C). Ventral shield of collar half-circle shaped. Anterior peristomial ring lobe triangular, exposed partially above the collar margin (Fig. 11D). Glandular ridge of chaetiger 2 narrow all around, it is whitish in unstained worms or stained with methyl green (Fig. 11A–B).

#### Taxon discussion

One specimen was found regenerating a radiolar crown. This species has been reported in the Pacific coast of Panama and some localities from Mexican Pacific (Tovar‐Hernández 2007).

### Notaulax californica

(Treadwell, 1906)

29E25A15-3D59-5944-8796-864BE1B6E027

Potamilla californica Treadwell 1906: 1178.
Hypsicomus sp.— Hartman 1942: 133.
Hypsicomus californicus .— Hartman 1956: 258, 262, 270; Hartman 1969: 701–702.
Notaulax californica .— Perkins 1984: 342–343, fig. 31.

#### Materials

- **Type status:** Other material. **Occurrence:** catalogNumber: LEMA-PO165; recordedBy: Beatriz Yáñez-Rivera; individualCount: 2; **Taxon:** phylum: Annelida; class: Polychaeta; order: Sabellida; family: Sabellidae; genus: Notaulax; **Location:** higherGeographyID: Pacific Ocean; higherGeography: Tropical Eastern Pacific; continent: America; islandGroup: Islas de Chamela; island: Isla Pajarera; country: México; countryCode: MX; stateProvince: Jalisco; municipality: La Huerta; maximumDepthInMeters: 7; verbatimLatitude: 19°33’22’’N; verbatimLongitude: 105°06’50’’W; **Identification:** identifiedBy: María Ana Tovar-Hernández; **Event:** samplingProtocol: Scuba dive; eventDate: June 26, 2013; year: 2013; month: 6; day: 26; habitat: On rock-coral; fieldNumber: Site 21; **Record Level:** language: Spanish; institutionID: Universidad de Guadalajara; collectionID: Colección Biológica del Laboratorio de Ecosistemas Marinos y Acuicultura; institutionCode: UDG; collectionCode: LEMA
- **Type status:** Other material. **Occurrence:** catalogNumber: LEMA-PO166; recordedBy: Beatriz Yáñez-Rivera; **Taxon:** phylum: Annelida; class: Polychaeta; order: Sabellida; family: Sabellidae; genus: Notaulax; **Location:** higherGeographyID: Pacific Ocean; higherGeography: Tropical Eastern Pacific; continent: America; islandGroup: Islas de Chamela; island: Isla Cocinas; country: México; countryCode: MX; stateProvince: Jalisco; municipality: La Huerta; minimumDepthInMeters: 3; maximumDepthInMeters: 4; verbatimLatitude: 19°32’57’’N; verbatimLongitude: 105°06’20’’W; **Identification:** identifiedBy: María Ana Tovar-Hernández; **Event:** samplingProtocol: Scuba dive; eventDate: June 27, 2013; year: 2013; month: 6; day: 27; habitat: On rock; fieldNotes: Site 16; **Record Level:** language: Spanish; institutionID: Universidad de Guadalajara; collectionID: Colección Biológica del Laboratorio de Ecosistemas Marinos y Acuicultura; institutionCode: UDG; collectionCode: LEMA

#### Description

Solitary fan worm associated with dead coral and rocks. Body length 11–14 mm, width 0.9–1.1 mm. Radiolar crown length 3–3.4 mm with 8–10 pairs of radioles. Thorax with eight chaetigers and abdomen with 60–67 chaetigers. Radioles with short bands of radiolar ocelli (Fig. 12A–B), each band as long as the space of 4–6 pinnules, ocelli distributed in single rows of 12 to 16 ocelli, bands located at three quarters of the radiolar crown length (Fig. 12C). Ventral margin of collar incised, forming rounded lappets. Base of radiolar crown (basal lamina or radiolar lobes) short, as long as the length of the first three segments in lateral view.

#### Taxon discussion

This is the first formal record in Mexico.

### Parasabella pallida

Moore, 1923

946B8803-459C-59CE-A373-3810EDAB4330

**Figure 5.**
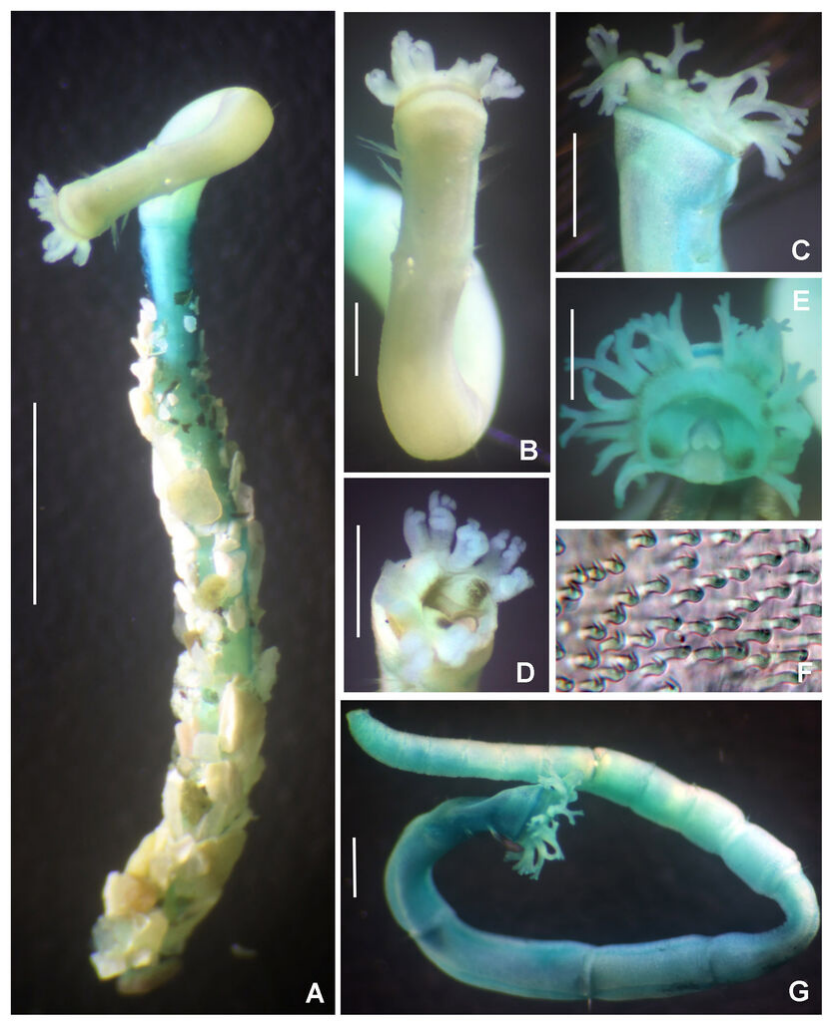
*Owenia collaris* Hartman, 1955. A) Worm partially inside its tube, B) anterior region and tentacular crown, ventral view, C) tentacular crown, lateral view, D-E) internal details of tentacular crown and pigmentation, F) neuropodial uncini, G) entire worm, removed tube. Scale bars: A) 2 mm, B–E, G) 0.5 mm, F) 1000x magnification.

**Figure 6.**
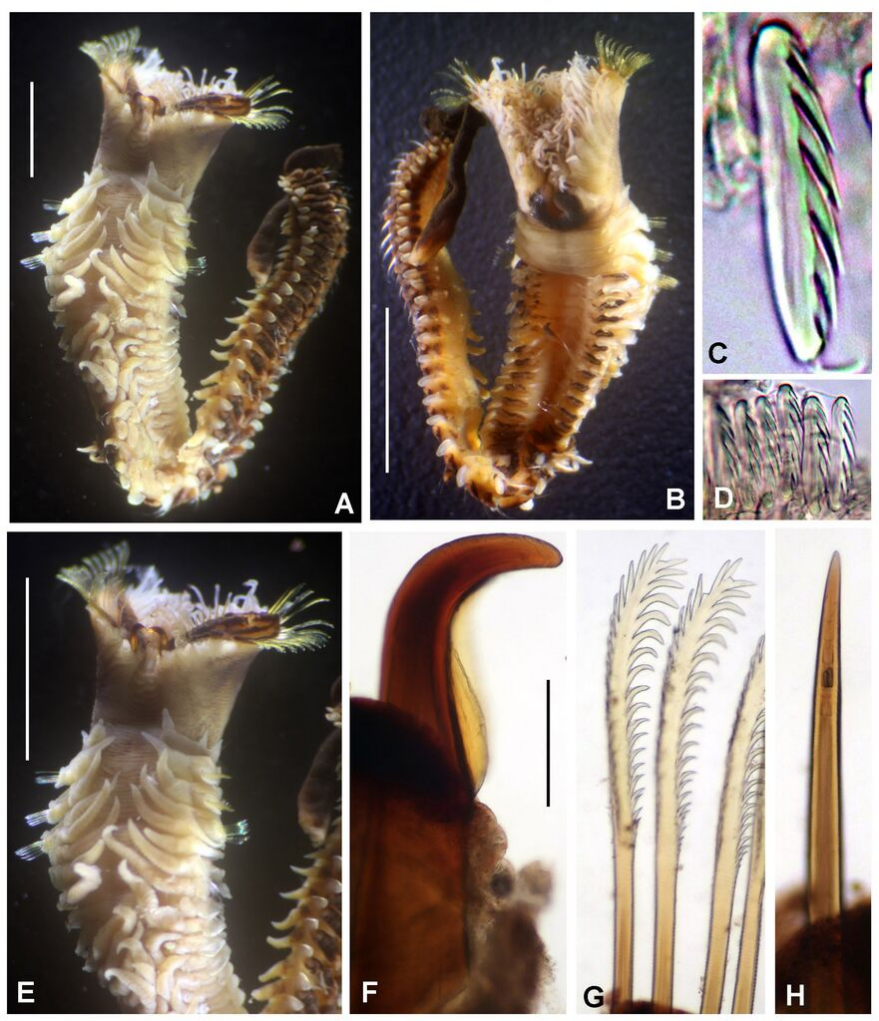
*Idanthyrsus cretus* Chamberlin, 1919. A) Entire worm, dorsal view, B) same, ventral view, C) abdominal uncinus dentition, lateral view, D) abdominal uncini, E) operculum and parathorax, F) nuchal hook, G) outer opercular paleae, H) inner opercular paleae. Scale bars: A) 2 mm, B, E) 4 mm, C) 1000x magnification, D, G–H) 400x magnification, F) 0.25 mm.

**Figure 7.**
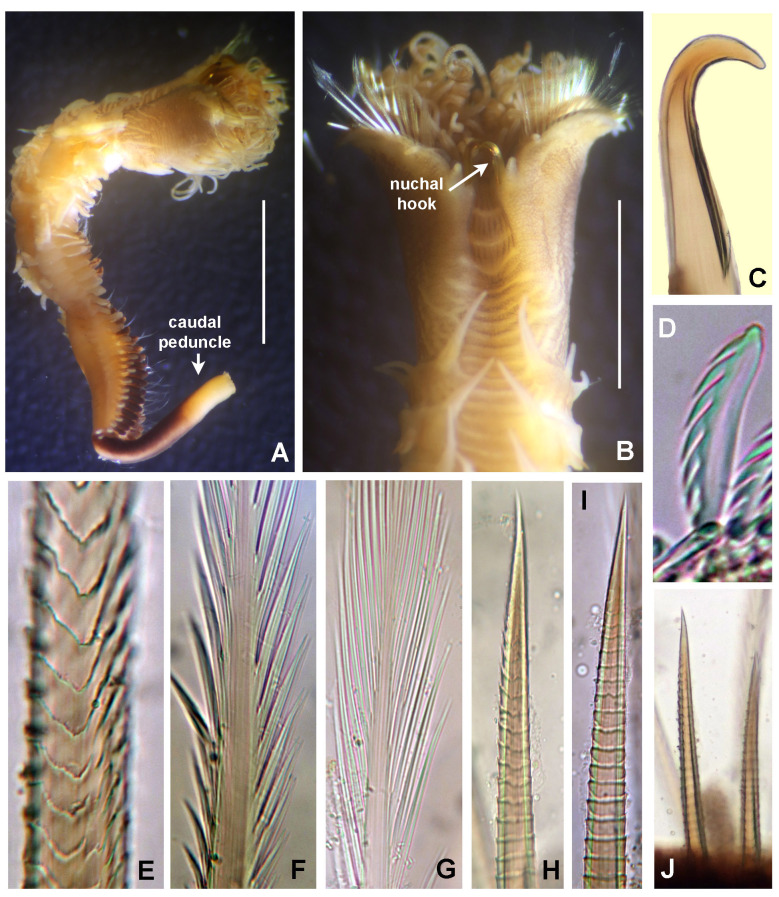
*Idanthyrsus mexicanus* Kirtley, 1994. A) Entire body, dorso-lateral view, B) operculum, dorsal view, C) nuchal hook, D) abdominal uncini, lateral view, E) base of outer opercular palea, F) mid-region of a outer opercular paleae, G) near distal end of outer opercular palea, H–J) inner opercular paleae. Scale bars: A) 3 mm, B) 1.5 mm, C, J) 400x magnification, D–I) 1000x magnification.

**Figure 8.**
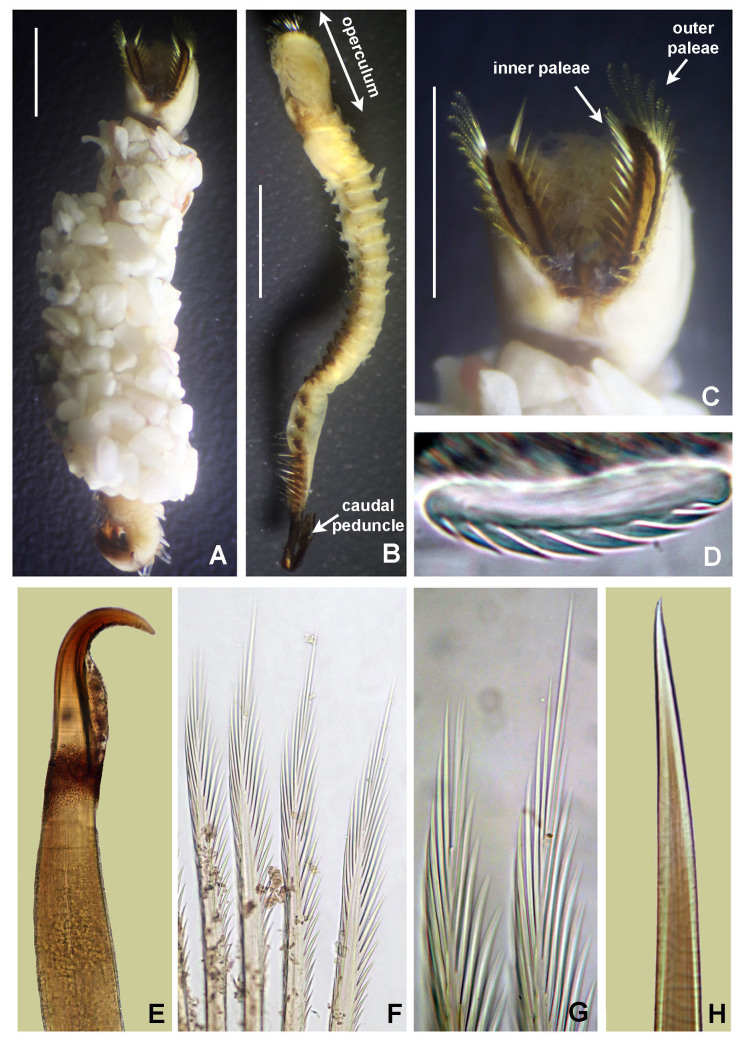
*Idanthyrsus* sp. A) Worm inside its tube, B) entire body, C) operculum, D) abdominal uncinus, E) nuchal hook, F) outer opercular paleae, G) radiolar tip of outer opercular palea, H) inner opercular palea. Scale bars: A, C) 2 mm, B) 3 mm, D, G–H) 1000x magnification, E–F) 400x magnification.

**Figure 9.**
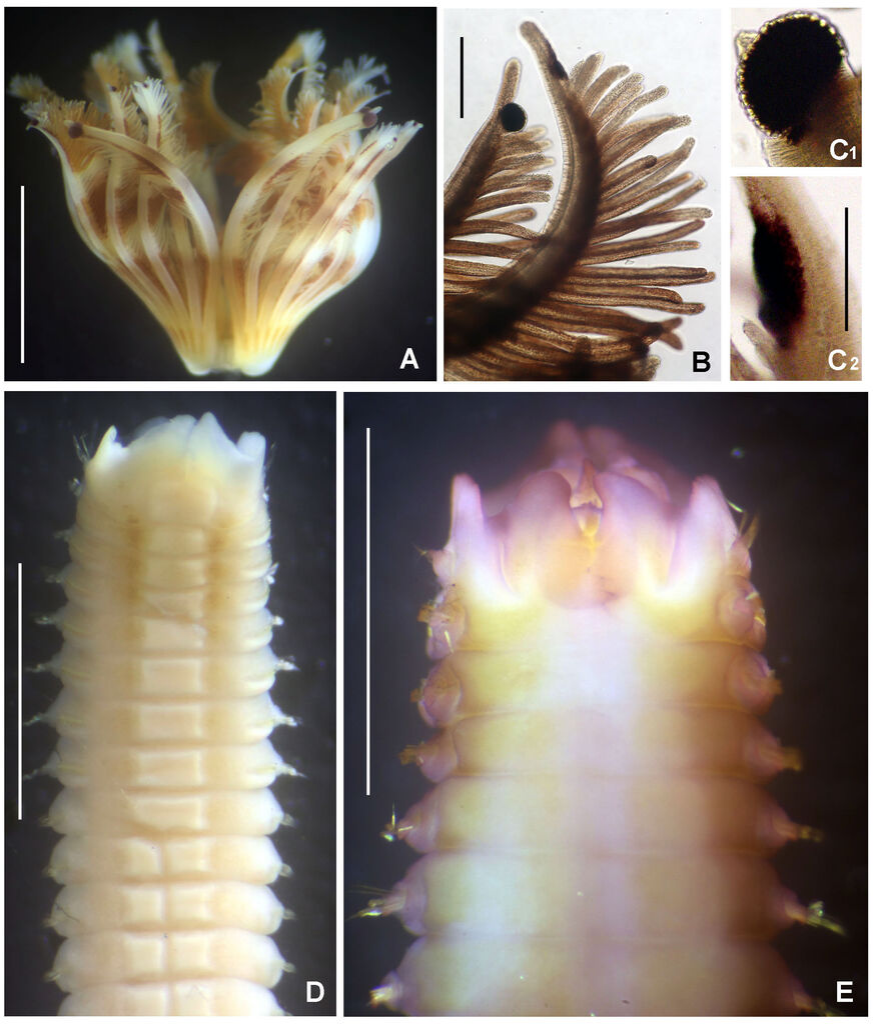
*Acromegalomma circumspectum* (Moore, 1923). A) Radiolar crown, dorsal view, B) compound radiolar eyes, C_1_) eye from dorsal-most radiole showing their ommatidia, C_2_) ocular spot from ventral-most radiole, D) thorax and anterior abdomen anterior, ventral view (crown removed), E) thorax, dorsal view (crown removed). Scale bars: A, D–E) 4 mm, B) 0.5 mm, C_1_–C_2_) 0.3 mm.

**Figure 10.**
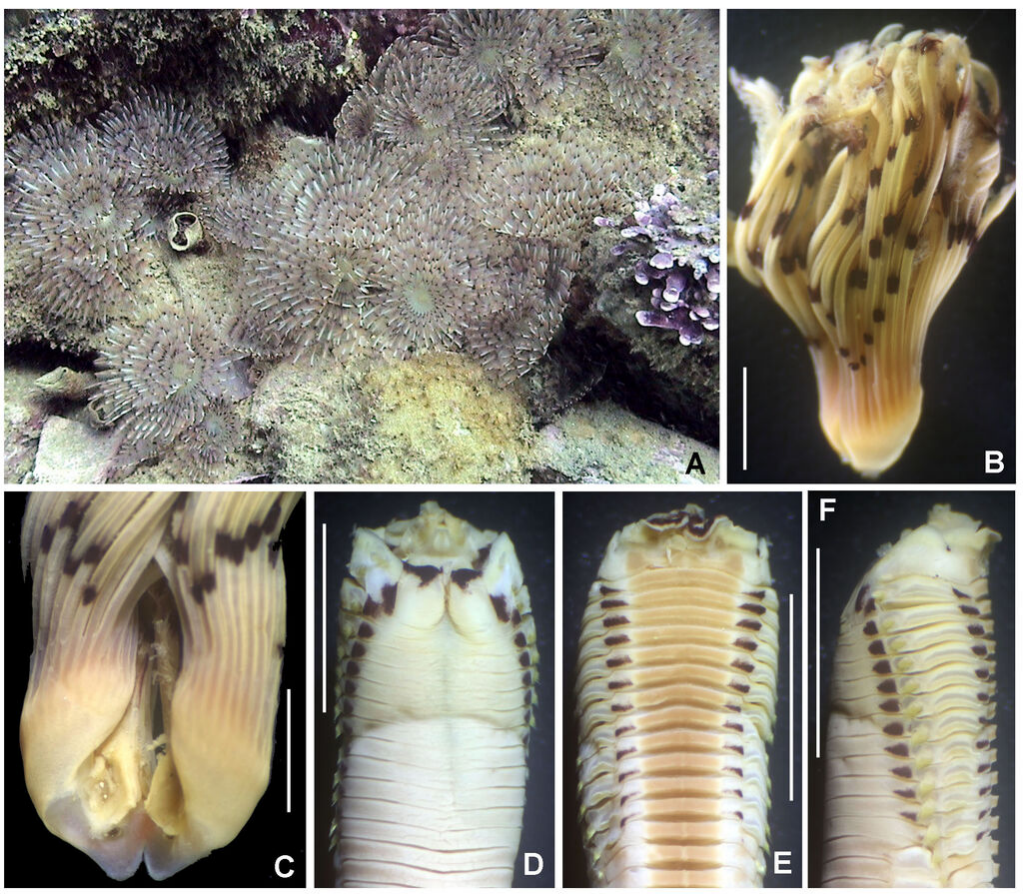
*Bispira monroi* (Hartman, 1961). A) Colony *in situ*, B) radiolar crown, C) base of crown, ventral view, D) thorax, dorsal view, E) same, ventral view, F) same, lateral view. Scale bars: A) not scaled, B, D–F) 5 mm, C) 2 mm.

**Figure 11.**
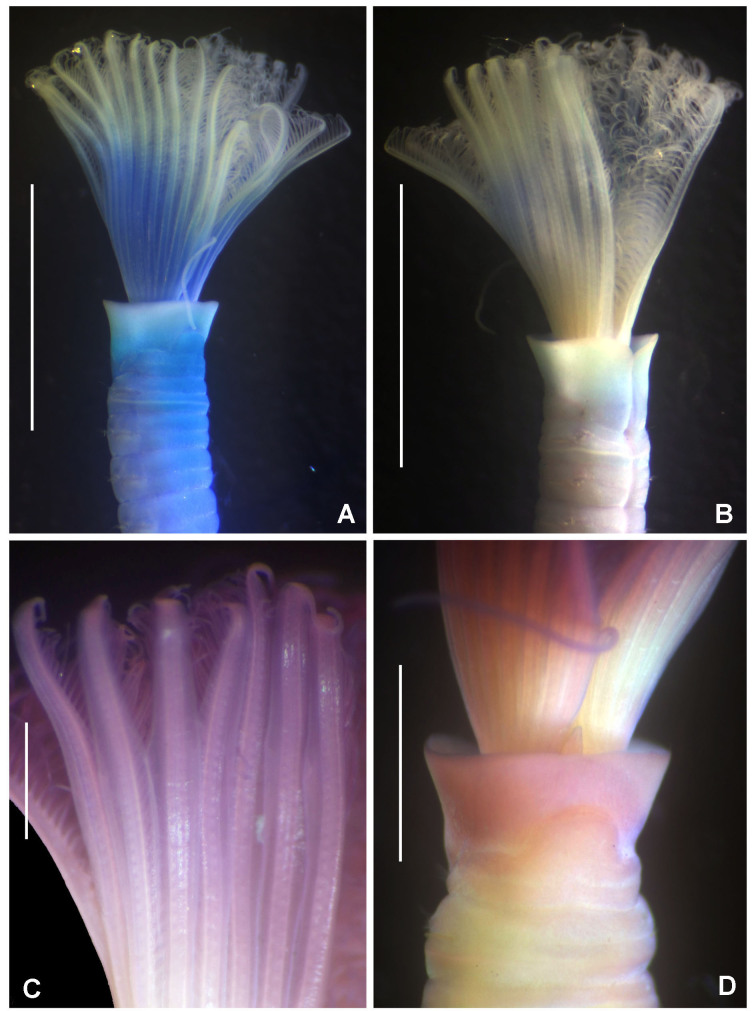
*Chone mollis* (Bush *in* Moore, 1904). A–B) Radiolar crown and first thoracic chaetigers, C) palmate membrane and radiolar flanges, D) collar and anterior peristomial ring lobe. A) Stained with methyl green, C–D) stained with Shirla-Stain A. Scale bars: A–B) 3.5 mm, C) 0.5 mm, D) 1 mm.

Parasabella pallida in *Moore 1923*: 241, 242.– Loi 1980: 144.— Bastida-Zavala et al. 2016: 407–408, figs. 2, 10D.— Tovar-Hernández et al. 2019: 5, fig. 2B.
Sabella media .– Hartman 1944b: 285 [in part, not pl. 23, fig. 42].
Demonax medius .– Hartman 1969: 675, 676 [in part, not figs. 1–5].
Demonax pallidus .– Perkins 1984: 313–315, figs. 15–16.— Tovar‐Hernández et al. 2009: 325–326, figs. 2b, f, 3c–d, 4c–e.

#### Materials

- **Type status:** Other material. **Occurrence:** catalogNumber: LEMA-PO167; recordedBy: Beatriz Yáñez-Rivera; individualCount: 1; **Taxon:** phylum: Annelida; class: Polychaeta; order: Sabellida; family: Sabellidae; genus: Parasabella; **Location:** higherGeographyID: Pacific Ocean; higherGeography: Tropical Eastern Pacific; continent: America; islandGroup: Islas de Chamela; island: Isla Cocinas; country: México; countryCode: MX; stateProvince: Jalisco; municipality: La Huerta; maximumDepthInMeters: 7; verbatimLatitude: 19°32’45’’N; verbatimLongitude: 105°06’27’’W; **Identification:** identifiedBy: María Ana Tovar-Hernández; **Event:** samplingProtocol: Scuba dive; eventDate: June 25, 2013; year: 2013; month: 6; day: 25; habitat: On rock-coral; fieldNumber: Site 15; **Record Level:** language: Spanish; institutionID: Universidad de Guadalajara; collectionID: Colección Biológica del Laboratorio de Ecosistemas Marinos y Acuicultura; institutionCode: UDG; collectionCode: LEMA

#### Description

**Figure 12.**
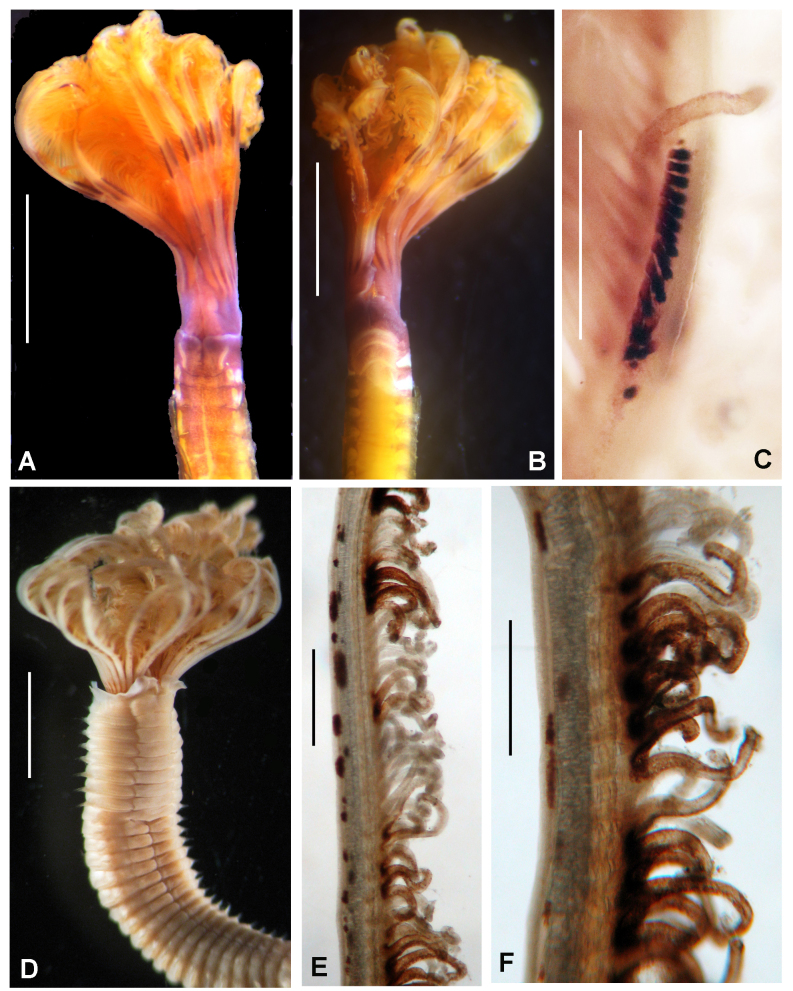
*Notaulax californica* (Treadwell, 1906) and *Parasabella pallida* Moore, 1923. *Notaulax californica*: A) crown and thorax, dorsal view, B) same, ventral view, C) radiolar ocelli. *Parasabella pallida*: D) crown, thorax and anterior abdomen, E–F) radiolar maculae. Scale bars: A–B, D) 2 mm, C, E–F) 0.3 mm.

Solitary fan worm with soft tubes, composed of fine sand and covered by algae and bryozoans. Body length 14–26 mm, 1.5–2.3 mm width. Radiolar crown length 6–10 mm with 12–14 pairs of radioles. Thorax with eight chaetigers and abdomen with 63–67 chaetigers. Radioles with brownish maculae (no eyes or ocelli) along radiolar length (Fig. 12D–F). Radiolar tips with broad flanges. Ventral shields well developed, contacting tori. Ventral collar margin incised, forming two rounded lappets with triangular tips.

#### Taxon discussion

Members of *Parasabella* seem like *Acromegalomma* at first view, but the latter have subdistal compound eyes in radioles (absent in *Parasabella*). Common in fouling from the Gulf of California (Tovar‐Hernández et al. 2009; Tovar-Hernández et al. 2019; Bastida-Zavala et al. 2016).

### Pseudobranchiomma schizogenica

Tovar-Hernández and Dean, 2014

EAA4594F-73B6-5264-A0CD-01B90C6CEFEF

Pseudobranchiomma schizogenica in Tovar-Hernández and Dean 2014: 936–944, figs. 1–5.— Keppel et al. 2019: 67–68, fig. 6.— Tovar-Hernández et al. 2019: 5, fig. 2C.

#### Materials

- **Type status:** Other material. **Occurrence:** catalogNumber: LEMA-PO168; recordedBy: Beatriz Yáñez-Rivera; individualCount: 3; sex: Hermaphrodite; lifeStage: Adult; reproductiveCondition: Ripe; **Taxon:** phylum: Annelida; class: Polychaeta; order: Sabellida; family: Sabellidae; genus: Pseudobranchiomma; **Location:** higherGeographyID: Pacific Ocean; higherGeography: Tropical Eastern Pacific; continent: America; islandGroup: Islas de Chamela; island: Isla Pajarera; country: México; countryCode: MX; stateProvince: Jalisco; municipality: La Huerta; maximumDepthInMeters: 9; verbatimLatitude: 19°33’22’’N; verbatimLongitude: 105°06’50’’W; **Identification:** identifiedBy: María Ana Tovar-Hernández; **Event:** samplingProtocol: Scuba dive; eventDate: June 27, 2013; year: 2013; month: 6; day: 27; habitat: On rock; fieldNumber: Site 21; **Record Level:** language: Spanish; institutionID: Universidad de Guadalajara; collectionID: Colección Biológica del Laboratorio de Ecosistemas Marinos y Acuicultura; institutionCode: UDG; collectionCode: LEMA

#### Description

**Figure 13.**
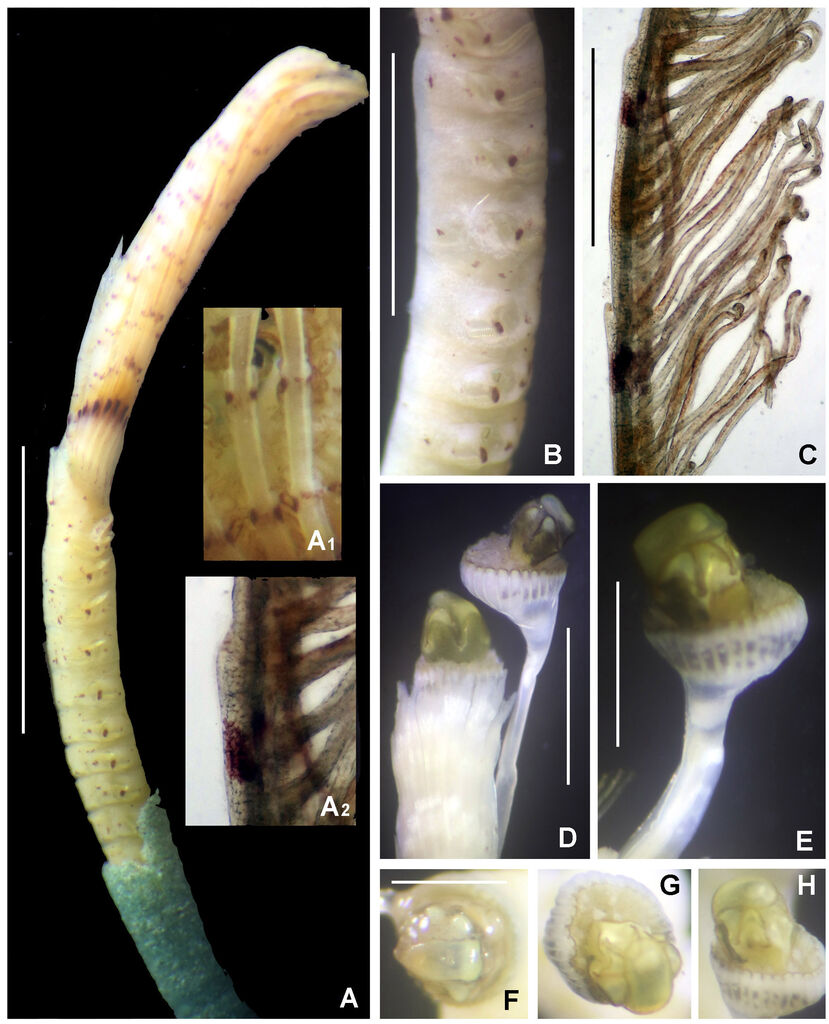
*Pseudobranchiomma schizogenica* Tovar-Hernández and Dean, 2014 and *Hydroides brachyacantha* Rioja, 1941a. *Pseudobranchiomma schizogenica*: A) radiolar crown and thorax, A_1_) paired radiolar eyes, A_2_) radiolar flanges, B) interramal eyes, C) radiole with flanges. *Hydroides brachyacantha*: D–H) opercula, different angle views. Scale bars: A–B) 3 mm, C, F–H) 0.3 mm, D–E) 0.5 mm.

Gregarious fan worms. Soft, thin and flexible tubes composed of fine sand. Body length 12–18 mm, 1 mm width. Radiolar crown 9–12 mm long with 8 pairs of radioles. Thorax with eight chaetigers and abdomen with 59–62 chaetigers. Base of branchial crown purple (Fig. 13A). Radioles with paired eyes along the entire length (Fig. 13A–A_1_). Radioles with paired flanges (Fig. 13A, C). Body with purple maculae (Fig. 13A) and interramal eyespots along the body, located between nopodium and neuropodium (Fig. 13A–B).

#### Taxon discussion

At first view, *Pseudobranchiomma* can be confused with *Branchiomma* because both are commonly gregarious, their bodies are full of purple maculae and both have interramal eyes and radioles with paired eyes. However, *Branchiomma* has long stylodes as tongue or straps-like filaments along radioles, easily seen under a stereoscope. In the Southern Gulf of California, it has been reported associated with man-made substrates in densities reaching 487 ind/m^2^, associated often with the invasive sabellid *Branchiomma bairdi* (Tovar-Hernández and Dean 2014). Both species have been reported as introduced in Australia (Capa and Murray 2015, Capa and Murray 2016) and both species have been also registered in Galapagos by Keppel et al. (2019): *B. bairdi* as introduced, whereas *P. schizogenica* inside their natural distribution area. As its reproduction is mainly by architomy (fission, asexual reproduction), it is common to find small specimens or clones in a chain below the parental worm (Tovar-Hernández and Dean 2014).

### Hydroides brachyacantha

Rioja, 1941

6097AAF2-2B76-55E3-8BAB-0D4C17DE15B0

Hydroides brachyacantha in *Rioja 1941a*: 169–172, pl. 3, fig. 2, pl. 4, figs. 1–9.— Sun et al. 2016: 49–54, figs. 5, 6A–F.— Bastida-Zavala et al. 2016: 413–414, figs. 3, 11B.— Chávez-López and Cruz-Gómez 2019: 162.
Hydroides brachyacanthus .– Bastida-Zavala and ten Hove 2003: 73–76, figs. 3A–M, 7A–F.– Çinar 2006: 225–226, fig. 2.– Bastida-Zavala 2008: 22–23, fig. 6C.— Tovar‐Hernández et al. 2009: 328–330, figs. 3j, 7d–f.

#### Materials

- **Type status:** Other material. **Occurrence:** catalogNumber: LEMA-PO169; recordedBy: Beatriz Yáñez-Rivera; individualCount: 2; **Taxon:** phylum: Annelida; class: Polychaeta; order: Sabellida; family: Sabellidae; genus: Hydroides; **Location:** higherGeographyID: Pacific Ocean; higherGeography: Tropical Eastern Pacific; continent: America; islandGroup: Islas de Chamela; island: Isla Cocinas; country: México; countryCode: MX; stateProvince: Jalisco; municipality: La Huerta; minimumDepthInMeters: 3; maximumDepthInMeters: 4; verbatimLatitude: 19°32’45’’N; verbatimLongitude: 105°06’27’’W; **Identification:** identifiedBy: María Ana Tovar-Hernández; **Event:** samplingProtocol: Scuba dive; eventDate: June 27, 2013; year: 2013; month: 6; day: 27; habitat: On coral; fieldNumber: Site 15; **Record Level:** language: Spanish; institutionID: Universidad de Guadalajara; collectionID: Colección Biológica del Laboratorio de Ecosistemas Marinos y Acuicultura; institutionCode: UDG; collectionCode: LEMA
- **Type status:** Other material. **Occurrence:** catalogNumber: LEMA-PO170; recordedBy: Beatriz Yáñez-Rivera; individualCount: 7; **Taxon:** phylum: Annelida; class: Polychaeta; order: Sabellida; family: Sabellidae; genus: Hydroides; **Location:** higherGeographyID: Pacific Ocean; higherGeography: Tropical Eastern Pacific; continent: America; islandGroup: Islas de Chamela; island: Isla Pajarera; country: México; countryCode: MX; stateProvince: Jalisco; municipality: La Huerta; maximumDepthInMeters: 7; verbatimLatitude: 19°33’22’’N; verbatimLongitude: 105°06’50’’W; **Identification:** identifiedBy: María Ana Tovar-Hernández; **Event:** samplingProtocol: Scuba dive; eventDate: June 26, 2013; year: 2013; month: 6; day: 26; habitat: On rock-coral; fieldNumber: Site 21; **Record Level:** language: Spanish; institutionID: Universidad de Guadalajara; collectionID: Colección Biológica del Laboratorio de Ecosistemas Marinos y Acuicultura; institutionCode: UDG; collectionCode: LEMA
- **Type status:** Other material. **Occurrence:** catalogNumber: LEMA-PO171; individualCount: 1; **Taxon:** phylum: Annelida; class: Polychaeta; order: Sabellida; family: Sabellidae; genus: Hydroides; **Location:** higherGeographyID: Pacific Ocean; higherGeography: Tropical Eastern Pacific; continent: America; islandGroup: Islas de Chamela; island: Isla Pajarera; country: México; countryCode: MX; stateProvince: Jalisco; municipality: La Huerta; minimumDepthInMeters: 3; maximumDepthInMeters: 4; verbatimLatitude: 19°33’29’’N; verbatimLongitude: 105°06’40’’W; **Identification:** identifiedBy: María Ana Tovar-Hernández; **Event:** samplingProtocol: Snorkel; eventDate: June 27, 2013; year: 2013; month: 6; day: 27; habitat: On coral; fieldNumber: Site 20; **Record Level:** language: Spanish; institutionID: Universidad de Guadalajara; collectionID: Colección Biológica del Laboratorio de Ecosistemas Marinos y Acuicultura; institutionCode: UDG; collectionCode: LEMA

#### Description

Body 8–10 mm long, 0.8–1 mm wide. Radiolar crown length 1.5–1.7 mm with 8–10 pairs of radioles. Thorax with seven chaetigers and abdomen with 61–64 segments. Verticil with 8-12 yellow to dark brown spines unequal in size (Fig. 13D–H). Dorsal hook broad, curved, larger than all other spines, covering central disc. Other spines with pointed tip and pronounced knob each. First and second pair of dorsal spines (lateral to dorsal hook) with tips and trunks wider than all other spines (Fig. 13D–H). Collar chaetae bayonet with two blunt teeth; distal blade smooth.

#### Taxon discussion

*Hydroides brachyacantha* Rioja, 1941 (Rioja 1941a), an important fouling serpulid species originally described from Mazatlán (Southern Gulf of California, Mexico) and Acapulco (southern Mexican Pacific), has been reported from the Mexican Pacific and numerous tropical and subtropical localities (Rioja 1941a, Tovar‐Hernández et al. 2009, Bastida-Zavala and ten Hove 2003, Bastida-Zavala 2008, Bastida-Zavala et al. 2016). At the Mazatlán Port, the mean annual density of *H. brachyacantha* during 2009 was 80 ind m^–2^ (fouling assemblages in metallic buoys), with a minimum of 4 ind m^–2^ in November and a maximum of 304 ind m^–2^ in March (Sun et al. 2016). Recently, a neotype was established by Sun et al. (2016), who also demonstrated that the previous records from the species in Australia belongs to a different lineage.

### Salmacina tribranchiata

Moore, 1923

044A1FB3-A5A2-5A73-9C43-FEE8B021728C

Filograna tribranchiata in Moore 1923: 250–251.
Salmacina dysteri tribranchiata .— Monro 1933: 1090–1091, Text-figure 31.— Berkeley and Berkeley 1941: 56.
Salmacina dysteri (not Huxley 1855).— Steinbeck and Ricketts 1941: 367.
Salmacina tribranchiata .— Rioja 1941b: 738–739, pl. 9, figs. 11–14.— Hartman 1961: 44.— Hartman 1969: 771–772, figs. 1–6.— Salazar-Vallejo and López-Muraira 1983: 111–112.— Bastida-Zavala 2008: 43, figs. 10H–J.— Bastida-Zavala et al. 2016: 433–434, figs. 8, 12E–F.

#### Materials

- **Type status:** Other material. **Occurrence:** catalogNumber: LEMA-PO172; recordedBy: Irving Ramírez-Santana; individualCount: 1; **Taxon:** phylum: Annelida; class: Polychaeta; order: Sabellida; family: Serpulidae; genus: Salmacina; **Location:** higherGeographyID: Pacific Ocean; higherGeography: Tropical Eastern Pacific; continent: America; islandGroup: Islas de Chamela; island: Isla Cocinas; country: México; countryCode: MX; stateProvince: Jalisco; municipality: La Huerta; verbatimDepth: 8; verbatimLatitude: 19°32’45’’N; verbatimLongitude: 105°06’27’’W; **Identification:** identifiedBy: Beatriz Yáñez-Rivera; **Event:** samplingProtocol: Scuba dive; eventDate: April 23, 2009; year: 2009; month: 4; day: 23; habitat: On coral; fieldNumber: Site 15; **Record Level:** language: Spanish; institutionID: Universidad de Guadalajara; collectionID: Colección Biológica del Laboratorio de Ecosistemas Marinos y Acuicultura; institutionCode: UDG; collectionCode: LEMA

#### Description

**Figure 14.**
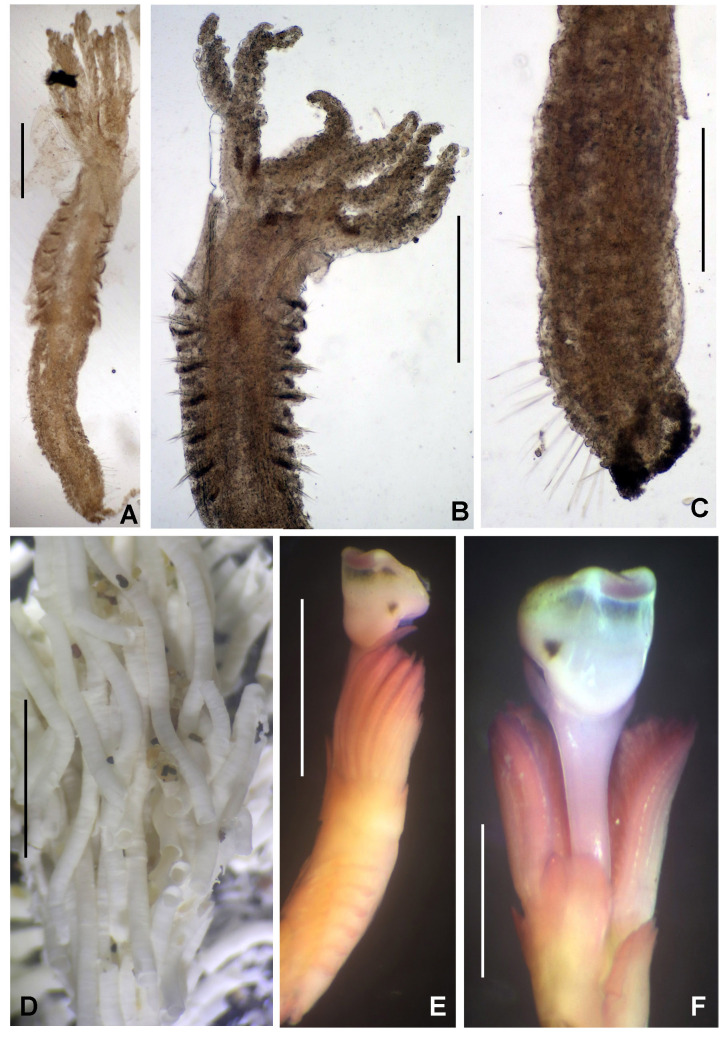
*Salmacina tribranchiata* (Moore, 1923) and *Spirobranchus minutus* Rioja, 1941b. *Salmacina tribranchiata*: A) Entire body, tube removed, B) radiolar crown and thorax, C) abdomen and pygidium, D) tubes. *Spirobranchus minutus*: E) radiolar crown and operculum, F) opercular peduncle and operculum. Scale bars: A–C) 0.2 mm, D) 2 mm, E) 1 mm, F) 0.5 mm.

Gregarious worm with tubes white, thin, with transversal ridges, lacking longitudinal ridges, peristomes or alveoli (D). Radiolar crown with 3–4 pairs of radioles (Fig. 14A). Opercular peduncle or operculum absent (Fig. 14B). Thorax with eigth chaetigers. Collar with fin- and blade-chaetae, with proximal denticulate expansion separate from the distal limbate zone, with 3–5 large teeth. Thoracic with “*Apomatus*” chaetae and thoracic uncini as rasp-shaped plates with 8–9 rows of teeth. Posterior abdomen with glandular areas (Fig. 14C).

#### Distribution

Widely reported in the Mexican Pacific (Bastida-Zavala 2008, Bastida-Zavala et al. 2016).

### Spirobranchus minutus

Rioja, 1941

011C9FFE-6A03-54AF-89C2-C51E15082F79

**Figure 15.**
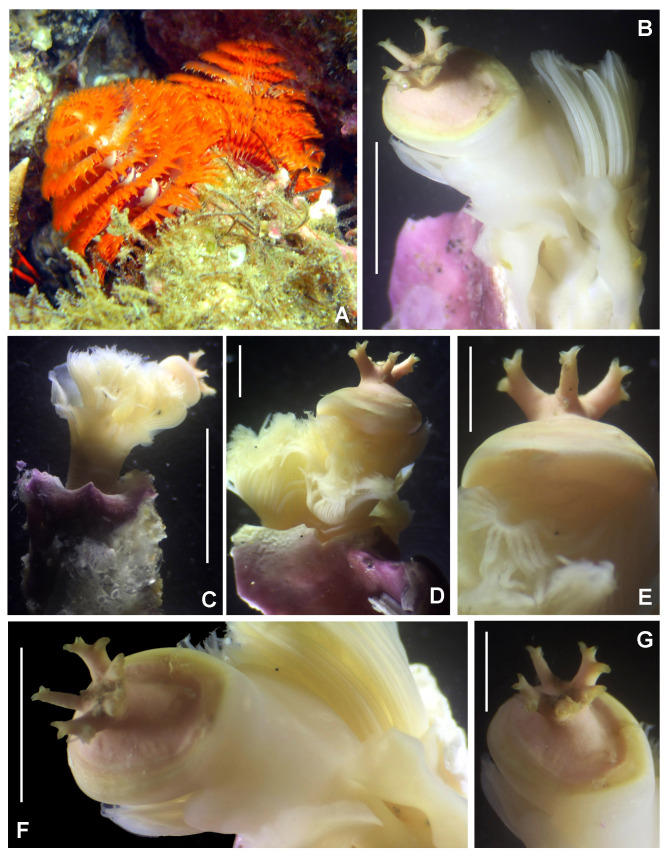
Spirobranchus cf. gaymardi sensu Bastida-Zavala (2008). A) Worm *in situ*, B, D–G) opercula, different angle views, C) radiolar crown and tube. Scale bars: A) not scaled, B, F) 6 mm, C) 5 mm, D–E, G) 3 mm.

Pomatoceros minutus in *Rioja 1941b*: 734–738, pl. 9, figs. 15–26.— Rioja 1942, 130–132, figs. 15-21.— Rioja 1947: 215.— Berkeley and Berkeley 1958: 405.— Bastida-Zavala 1993: 35.— Bastida-Zavala 2008: 31–33, figs. 7H–M.
Spirobranchus minutus .— Pillai 2009: 146–148.— Bastida-Zavala et al. 2016: 435–437, figs 8, 13H.

#### Materials

- **Type status:** Other material. **Occurrence:** catalogNumber: LEMA-PO173; recordedBy: Beatriz Yáñez-Rivera; individualCount: 1; **Taxon:** phylum: Annelida; class: Polychaeta; order: Sabellida; family: Sabellidae; genus: Spirobranchus; **Location:** higherGeographyID: Pacific Ocean; higherGeography: Tropical Eastern Pacific; continent: America; islandGroup: Islas de Chamela; island: Isla Cocinas; country: México; countryCode: MX; stateProvince: Jalisco; municipality: La Huerta; minimumDepthInMeters: 3; maximumDepthInMeters: 4; verbatimLatitude: 19°32’45’’N; verbatimLongitude: 105°06’27’’W; **Identification:** identifiedBy: María Ana Tovar-Hernández; **Event:** samplingProtocol: Scuba dive; eventDate: June 27, 2013; year: 2013; month: 6; day: 27; habitat: On coral; fieldNumber: Site 15; **Record Level:** language: Spanish; institutionID: Universidad de Guadalajara; collectionID: Colección Biológica del Laboratorio de Ecosistemas Marinos y Acuicultura; institutionCode: UDG; collectionCode: LEMA

#### Description

Body 4.2 mm long, 0.5 mm wide. Thorax with seven chaetigers and abdomen +5 (incomplete specimen). Opercular peduncle with thin distal wings. Operculum hoof-shaped, calcareous, white and dark spots basally on each side and anterior dark (Fig. 14E–F). Thoracic membrane extends to last thoracic chaetiger. Collar with limbate chaetae.

#### Distribution

Widely reported along the Mexican Pacific (Bastida-Zavala et al. 2016).

### Spirobranchus 123gaymardi

(de Quatrefages, 1866)

2624A5C4-BB4F-5D53-BDB7-F027F31F151D

Cymospira gaymardi in de Quatrefages 1866: 539–540, pl. 20, fig. 13.
Spirobranchus gaymardi .— Fiege and ten Hove 1999: 356–362, figs. 1–3.
Spirobranchus cf. gaymardi .— Bastida-Zavala 2008: 48, fig. 12A–B.

#### Materials

- **Type status:** Other material. **Occurrence:** catalogNumber: LEMA-PO174; recordedBy: Beatriz Yáñez-Rivera; individualCount: 2; lifeStage: Adult; **Taxon:** phylum: Annelida; class: Polychaeta; order: Sabellida; family: Serpulidae; genus: Spirobranchus; **Location:** higherGeographyID: Pacific Ocean; higherGeography: Tropical Eastern Pacific; continent: America; islandGroup: Islas de Chamela; island: Isla Cocinas; country: México; countryCode: MX; stateProvince: Jalisco; municipality: La Huerta; maximumDepthInMeters: 7; verbatimLatitude: 19°32’57’’N; verbatimLongitude: 105°06’27’’W; **Identification:** identifiedBy: María Ana Tovar-Hernández; **Event:** samplingProtocol: Scuba dive; eventDate: June 25, 2013; year: 2013; month: 6; day: 25; habitat: On coral; fieldNumber: Site 16; **Record Level:** language: Spanish; institutionID: Universidad de Guadalajara; collectionID: Colección Biológica del Laboratorio de Ecosistemas Marinos y Acuicultura; institutionCode: UDG; collectionCode: LEMA

#### Description

Tube with a prominent longitudinal ridge and a robust spine extending over the tube mouth (Fig. 15C–D). Entire worms 26–30 mm long, 5–6 mm wide. Operculum calcareous, pinkish, oval-shaped 4 mm x 6 mm. Opercular peduncle with wide wings. Opercular plate with three short spines emerging from a short common stem, with 2–3 secondary spinules on each (Fig. 15E–G). Red radioles, in spiral arrangement with six whorls (Fig. 15A). Thoracic membrane extends to last thoracic chaetiger, forming a short ventral apron.

#### Taxon discussion

Widely distributed in Mexican Pacific (Bastida-Zavala 2008). Bastida-Zavala (2008) reports only three wide spines on the opercular plate of S. cf. gaymardi in comparison with the five spines in specimens from Bahía de Chamela. *Spirobranchus spinosus* Hartman have five spines, but specimens from Bahía de Chamela cannot be attributed to that taxon, based on the following differences: in *S. spinosus*, the spines are thinner than in S. cf. gaymardi; each spine has 5–8 spinules versus 2–3 spinules in S. cf. gaymardi; each spines is separated one from the other, whereas spines emerge from the short common stem in S. cf. gaymardi. Consequently, the number of spines cannot be used to discern between S. cf. gaymardi and *S. spinosus* as suggested in some taxonomic keys.

## Discussion

It is notable that the most important inventories of some of the main marine taxonomic groups in the Bay have been carried out in the last 10 years, since 2009, so that the present study provides a first approach to the polychaete tubeworm worms of Bahía de Chamela, including a new record for Mexico and the establishment of a new taxon. Lack of knowledge of Annelida, as in other marine invertebrates, is common in most of the Mexican tropical Pacific. This knowledge is particularly important in marine-protected areas, since species records are a critical tool to generate a complete fauna list as a first step to understand distribution patterns, carry out subsequent monitoring of biodiversity and finally to assess the effectiveness of protected areas. However, this information should include accurate curatorial data from sampling technique, habitat, distribution notes along with preservation and proper storage in voucher collections. The present study provided all these data and could stimulate further research on the different groups of polychaete annelids in the Bay.

Amongst the families here reported, maldanids, also known as bamboo worms, are discretely mobile, deposit feeders, inhabiting usually soft sediments (Jumars et al. 2015). Tubes of *Clymenura scutata* sp. n. and *Isocirrus tropicus* were found attached to rock surfaces, the latter being also found in sand.

Oweniids, although mostly tubicolous, are considered discretely mobile because they can extend and move their tubes within sediment. These worms are primarily surface deposit feeders, but some species with tentacular crowns also suspension feed (Jumars et al. 2015). *Owenia collaris* have tentacular crowns and were found in sand.

Sabellariids, also known as honeycomb, sand-castle or sand-mason worms, usually build their tubes either attached to hard substrata or to other sabellariid tubes, in some cases forming massive reefs, but some species are solitary (Jumars et al. 2015, Helm et al. 2018). In Bahía de Chamela, *Idanthyrsus cretus* and *I. mexicanus* were found attached to rocks and corals, while the potential new species of *Idanthyrsus* (here refered to as *Idanthyrsus* sp.) after *Chávez-López (2019)*) was found amongst tubes of *I. cretus*.

Sabellids, known as feather-duster worms, fan worms or sea flowers, are sessile, tube builders. They inhabit tubes that they build with secreted mucus and attached mud or sand particles, except *Glomerula* that builds a calcareous tube (Capa et al. 2019). Species can be solitary or gregarious. Their members are mixed suspension feeders largely dependent upon bottom currents to bring particles within range of their downstream collecting systems (Jumars et al. 2015, Capa et al. 2019). In Bahía de Chamela, *Acromegalomma circumspectum*, *Chone mollis*, *Notaulax californica* and *Parasabella pallida* were found to be solitary and *Bispira monroi* and *Pseudobranchiomma schizogenica* are gregarious.

Serpulidae includes worms known as Christmas tree worms. All serpulids secret calcareous tubes and they are sessile suspension feeders (Jumars et al. 2015, Kupriyanova et al. 2019). All species here reported (*Hydroides brachyacantha*, *Salmacina tribranchiata*, *Spirobranchus minutus* and S. cf. gaymardi) were found in dead corals.

In the Mexican Pacific coast, several species of introduced polychaete worms of Sabellidae and Serpulidae have been reported, mainly associated with man-made substrates, such as dock pilings, boat hulls, buoys, ropes and aquaculture infrastructure (Bastida-Zavala 2008, Bastida-Zavala et al. 2016, Tovar‐Hernández et al. 2009, Tovar-Hernández et al. 2014, del Pasqua et al. 2018). Both families, Sabellidae and Serpulidae, are two of the most important groups of worms that travel on ship’s hulls and ballast water or attached to cultured molluscs or parasitising them. Thus, several species have been translocated outside the natural distribution range, generating, in some cases, ecological and economic negative impacts in their arrival localities (Capa et al. 2019, Kupriyanova et al. 2019). Introduced species were not detected in Bahía de Chamela, but their presence cannot be discarded since all specimens reviewed in the present study proceed from natural substrates. Consequently, a specific monitoring programme is desirable for the early detection of harmful tubicolous worms in that natural protected area.

## Supplementary Material

XML Treatment for Clymenura scutata

XML Treatment for Owenia collaris

XML Treatment for Isocirrus tropicus

XML Treatment for Idanthyrsus cretus

XML Treatment for Idanthyrsus mexicanus

XML Treatment for Idanthyrsus

XML Treatment for Acromegalomma circumspectum

XML Treatment for Bispira monroi

XML Treatment for Chone mollis

XML Treatment for Notaulax californica

XML Treatment for Parasabella pallida

XML Treatment for Pseudobranchiomma schizogenica

XML Treatment for Hydroides brachyacantha

XML Treatment for Salmacina tribranchiata

XML Treatment for Spirobranchus minutus

XML Treatment for Spirobranchus 123gaymardi

## Figures and Tables

**Table 1.** Occurrence record of tubicolous worms (Annelida, Polychaeta) in Chamela islands.

Infraclass	Family	Genus	Species	Taxonomic author	Voucher	Site 7	Site 14	Site 15	Site 16
Scolecida	Maldanidae	* Clymenura *	*Clymenura scutata*	Tovar-Hernández and Yáñez-Rivera, 2020	LEMA-PO153, UANL 8144, ICML-EMU 12758		1		1
Scolecida	Maldanidae	* Isocirrus *	*Isocirrus tropicus*	(Monro 1928)	LEMA-PO154		1		
Incerta sedis	Oweniidae	* Owenia *	*Owenia collaris*	Hartman 1955	LEMA-PO155		1		
Canalipalpata	Sabellariidae	* Idanthyrsus *	*Idanthyrsus cretus*	Chamberlin 1919	LEMA-PO156, PO157		1		
Canalipalpata	Sabellariidae	* Idanthyrsus *	*Idanthyrsus mexicanus*	Kirtley 1994	LEMA-PO158		1		
Canalipalpata	Sabellariidae	* Idanthyrsus *	*Idanthyrsus* sp.	Chávez-López 2019	LEMA-PO159	1			
Canalipalpata	Sabellidae	* Acromegalomma *	*Acromegalomma circumspectum*	(Moore 1923)	LEMA-PO160, PO161	1		1	
Canalipalpata	Sabellidae	* Bispira *	*Bispira monroi*	(Hartman 1961)	LEMA-PO162		1		
Canalipalpata	Sabellidae	* Chone *	*Chone mollis*	(Bush 1904)	LEMA-PO163, PO164		1		1
Canalipalpata	Sabellidae	* Notaulax *	*Notaulax californica*	(Treadwell 1906)	LEMA-PO165, PO166		1		1
Canalipalpata	Sabellidae	* Parasabella *	*Parasabella pallida*	Moore 1923	LEMA-PO167		1		
Canalipalpata	Sabellidae	* Pseudobranchiomma *	*Pseudobranchiomma schizogenica*	Tovar-Hernández and Dean 2014	LEMA-PO168	1			
Canalipalpata	Serpulidae	* Hydroides *	*Hydroides brachyacantha*	Rioja 1941a	LEMA-PO169, PO170, PO171		1		1
Canalipalpata	Serpulidae	* Salmacina *	*Salmacina tribranchiata*	(Moore 1923)	LEMA-PO172		1		
Canalipalpata	Serpulidae	* Spirobranchus *	*Spirobranchus minutus*	Rioja 1941b	LEMA-PO173		1		
Canalipalpata	Serpulidae	* Spirobranchus *	Spirobranchus cf. gaymardi	(de Quatrefages 1866)	LEMA-PO174		1		

**Table 2.** Main features of some *Clymenura* species.

Species	Prostomial eyes	Cephalic plate	Dentition of uncini in first thoracic chaetiger	Pre-anal achaetous segments	Cirri of anal plaque	Distribution (Marine Provinces according to Spalding et al. 2007)
*C. cirrata* (Ehlers, 1887)	? Not described by Ehlers (1887) *^1^	Notched laterally	Marked *^2^	1	four long cirri	Tropical North-western Atlantic
*C. columbiana* (Berkeley, 1929)	Present	Notched lateral and dorsally	Reduced	3	20 short cirri and a long mid-ventral cirrus	Cold Temperate Northeast Pacific
*C. snaiko* Read, 2011	Absent	Notched laterally	Marked	2	16 short cirri and mid-ventral cirri longest	Southern New Zealand
*C. scutata* sp. nov.	Absent	Entire	Marked	2	28 alternating short and long cirri and a long mid-ventral cirrus	Tropical East Pacific

## Footnotes

*1 Neither mentioned in Verrill (1900, page 654-655) and Read (2011, page 44).

*2 Ehlers (1887, page 23, plate 46, fig. 13) described and illustrated a thoracic uncini with four marked teeth, but there is no indication if it corresponds to the first segment. Later, Verrill (1900, page 654) described uncini from the first chaetiger with a marked dentition. Read (2011, pages 44) codified this feature as “n” (not reduced)
